# A Historical Overview of the Classification, Evolution, and Dispersion of *Leishmania* Parasites and Sandflies

**DOI:** 10.1371/journal.pntd.0004349

**Published:** 2016-03-03

**Authors:** Mohammad Akhoundi, Katrin Kuhls, Arnaud Cannet, Jan Votýpka, Pierre Marty, Pascal Delaunay, Denis Sereno

**Affiliations:** 1 Service de Parasitologie-Mycologie, Hôpital de l’Archet, Centre Hospitalier Universitaire de Nice, Nice, France; 2 Division of Molecular Biotechnology and Functional Genetics, Technical University of Applied Sciences Wildau, Wildau, Germany; 3 Inserm U1065, Centre Méditerranéen de Médecine Moléculaire, Université de Nice-Sophia Antipolis, Nice, France; 4 Biology Centre, Institute of Parasitology, Czech Academy of Sciences, Prague, Czech Republic; 5 Department of Parasitology, Faculty of Science, Charles University in Prague, Prague, Czech Republic; 6 MIVEGEC, UMR CNRS-IRD-Université de Montpellier Centre IRD, Montpellier, France; 7 UMR177, Centre IRD de Montpellier, Montpellier, France; Institut de Recherche pour le Développement, FRANCE

## Abstract

**Background:**

The aim of this study is to describe the major evolutionary historical events among *Leishmania*, sandflies, and the associated animal reservoirs in detail, in accordance with the geographical evolution of the Earth, which has not been previously discussed on a large scale.

**Methodology and Principal Findings:**

*Leishmania* and sandfly classification has always been a controversial matter, and the increasing number of species currently described further complicates this issue. Despite several hypotheses on the origin, evolution, and distribution of *Leishmania* and sandflies in the Old and New World, no consistent agreement exists regarding dissemination of the actors that play roles in leishmaniasis. For this purpose, we present here three centuries of research on sandflies and *Leishmania* descriptions, as well as a complete description of *Leishmania* and sandfly fossils and the emergence date of each *Leishmania* and sandfly group during different geographical periods, from 550 million years ago until now. We discuss critically the different approaches that were used for *Leishmana* and sandfly classification and their synonymies, proposing an updated classification for each species of *Leishmania* and sandfly. We update information on the current distribution and dispersion of different species of *Leishmania* (53), sandflies (more than 800 at genus or subgenus level), and animal reservoirs in each of the following geographical ecozones: Palearctic, Nearctic, Neotropic, Afrotropical, Oriental, Malagasy, and Australian. We propose an updated list of the potential and proven sandfly vectors for each *Leishmania* species in the Old and New World. Finally, we address a classical question about digenetic *Leishmania* evolution: which was the first host, a vertebrate or an invertebrate?

**Conclusions and Significance:**

We propose an updated view of events that have played important roles in the geographical dispersion of sandflies, in relation to both the *Leishmania* species they transmit and the animal reservoirs of the parasites.

## Introduction

Leishmaniases are vector-borne diseases caused by obligate protozoan parasites from the genus *Leishmania* (Trypanosomatida: Trypanosomatidae). Leishmaniases are endemic in large areas of the tropics, subtropics, and the Mediterranean basin, including more than 98 countries, where there are a total of 350 million people at risk and 12 million cases of infection. Canine leishmaniasis is a serious problem, and it is estimated that 2.5 million dogs are infected in the Mediterranean basin only [1]. Among the endemic regions on five continents, there is an estimated incidence of 0.7–1.2 million cases of cutaneous leishmaniasis (CL) and 0.2–0.4 million cases of visceral leishmaniasis (VL) in these countries [2]. The disease is absent in New Zealand and the southern Pacific. Leishmaniasis is transmitted by the bite of infected female sandflies, whose hosts are animals such as canids, rodents, marsupials, hyraxes, or human beings. Approximately 53 *Leishmania* species have been described (without considering the synonyms and including all five subgenera and complexes: *Leishmania*, *Viannia*, *Sauroleishmania*, *L*. *enriettii* complex, and *Paraleishmania*); of these, 31 species are known to be parasites of mammals and 20 species are pathogenic for human beings. *Leishmania* parasites cause four main clinical forms of the disease—according to the location of the parasite in mammalian tissues—referred to as visceral, cutaneous, diffuse cutaneous, and mucocutaneous leishmaniasis. The most common form is cutaneous disease, and the ten countries of Afghanistan, Algeria, Colombia, Brazil, Iran, Syria, Ethiopia, North Sudan, Costa Rica, and Peru together account for 70% to 75% of the global estimated CL incidence [2]. Regarding visceral leishmaniasis, more than 90% of all cases occur in just the six countries of India, Bangladesh, Sudan, South Sudan, Brazil, and Ethiopia [2]. Leishmaniasis currently constitutes a major global public health problem, showing an increasing burden over the last decade [2].

Leishmaniasis has a long history, dating to 2,500 B.C., with several primitive descriptions of the disease having been found in ancient writings and recent molecular findings from ancient archeological material. A detailed history of *Leishmania* descriptions is gven in Table 1.

**Table 1:** History of *Leishmania* descriptions.

Century	Author (Year): Description
B.C.	(2,500 to 1,500 B.C.): First description of conspicuous lesions similar to current cutaneous leishmanisis (CL) lesions. (2,000 B.C.): *Leishmania donovani* infection in ancient Egyptian and Christian Nubian mummies. (1,500 B.C.): Report of *Leishmania* DNA in northern Sudan. (800 B.C.): *Leishmania* infection in a 6-year-old girl mummy in Peru. (700 B.C.): Similar descriptions of CL discovered on tablets from King Ashurbanipal. (650 B.C.): Records of what seems to be CL in the Tigris–Euphrates basin.
A.D.	(First century A.D.): Evidence for the presence of the cutaneous form of the disease in Ecuador and Peru, South America. Avicenna (10th century A.D.): Description of cutaneous lesions called Balakh sore and probability of mosquito intervention. (15th and 16th centuries A.D.: Inca period): Notification of "valley sickness," "Andean sickness," or "white leprosy," which are likely to be South American CL.
18th century	Russell (1756): First detailed clinical description of the disease. Indian physicians (1756): Description of kala azar clinical symptoms (kālā āzār: kālā meaning black and āzār meaning fever or disease). Cosme Bueno (1764): First suspicions reporting the probable role of phlebotomine sandflies in disease transmission in the New World.
19th century	Villar (1859): Earliest traceable clinical description of the Peruvian ‘‘uta,” similar to the "Aleppo button." Borovsky (1898): First accurate description of the causative agent of the oriental sore, reference to Protozoa.
first half of 20th century	Leishman (1901): Identification of organisms, as "trypanosomes," in smears from the spleen of an Indian patient deceased from "dum-dum fever." Donovan (1901): Confirms the presence of what became known as Leishman-Donovan bodies in the smears from Indian patients. First description of the link between Leishman-Donovan bodies and kala azar. Ross (1903): Proposed the name of *Leishmania donovani* for the Leishman-Donovan bodies. Wright (1903): Description of *Helcosoma tropica* (*L*. *tropica*). Leishman and Rogers (1904): Demonstrated oval intracellular amastigotes can differentiate into flagellated promastigotes. Rogers (1904): First successful in vitro cultivation of the flagellated forms. Laveran and Chatoin (1904): First case of kala azar in the Mediterranean region. Sergent and colleagues (1905): First report of CL transmited by sandflies of the *Phlebotomus* genus. Patton (1907): Evidence of the presence of Leishman-Donovan bodies in peripheral blood lymphocytes and its flagellated forms in the sandfly's gut. Nicolle (1908): Isolation of *Leishmania* parasites from a child or "infant," leading to name *Leishmania infantum*. Differentiation between the Mediterranean visceral leishmaniasis caused by *L*. *infantum* and the Indian kala azar due to *L*. *donovani*. Nicolle and Comple (1908): Isolation of *Leishmania* parasites from infected dogs. Lindenberg, Carini, and Paranhos (1909): Confirm the presence of autochthonous cutaneous leishmaniasis, "Baurú ulcer," in the Americas. Wenyon (1911): Incrimination of *Phlebotomus* as the probable vector of diseases caused by *Leishmania* in the Old World. Splendore (1911): *Leishmania* as the causative agent of mucocutaneous lesions "Espundia." Vianna (1911): Description of *L*. *braziliensis*. Migone (1913): First report of visceral leishmaniasis in the Americas. Yakimoff and Schokhor (1914): Proposition of the names *L*. *tropica minor* and *L*. *tropica major* to separate parasites causing "dry urban" and "wet rural" cutaneous leishmaniasis. Casteliani and Chalmers (1919): *L*. *donovani archibaldi* as the ethiological agent of a lethal form of visceral leishmaniasis. Aragão (1922): Reproduced in a dog the clinical signs of leishmaniasis by injecting squashed infected sandflies. Montenegro (1923): Experimental inoculation of *L*. *braziliensis*, introduction of the intradermal test (Montenegro skin test), still in use for the diagnosis of leishmaniasis. Penna (1934): First record of the Amazonian visceral leishmanaisis. Chagas (1936): Description of visceral leishmaniasis in Brazil. Cunha and Chagas (1937): Isolation of *L*. *chagasi* from Brazilian VL. Swaminath and colleagues (1942): Demontrated the process of *Leishmania* transmition to humans by sandflies using a group of volunteers. Hoare (1948): Demonstrated the *Leishmania* circulation in sandflies, indicating the flagellates being set free and multiplying in the sandfly intestine; the infection later is caused through the posterior station (like *Trypanosoma cruzi*). Kirk (1949): Classification of *Leishmania* according to their morphology, culture characteristics, clinical and epidemiological aspects of infections in human and other natural hosts, cross-immunity, serological tests, and xenodifferentiation. Propose a complete nomenclature of the *Leishmania* genus and their synonyms.
second half of 20th century	Biagi (1953): Discription of various *Leishmania* species. Pessôa (1961): Present the first list of known *Leishmania* species in the Americas. Use of the trinomial system for *Leishmania*. Adler (1962): Reports transient cryptic infections in mice by *L*. *adleri*, which usually infects lizards, that lead to the proposal of the evolution of *Leishmania* species infecting mammals from reptilian parasites. Adler (1963 and 1964): Differentiates *L*. *tropica*, *L*. *mexicana*, and *L*. *braziliensis* with serological techniques. Proposed a taxonomy for *Leishmania* infecting hummans and lizards. Shaw (1964): Demostrates the transmission of *Endotrypanum schaudinni* by *Phlebotomus* species. Hoare and Wallace (1966): Introduced new terms for the description of the *Leishmania* developmental stages. Lainson and Shaw (1970): Subdivide *Leishmania* species into two groups: "fast-growing (*L*. *mexicana*)" and "slow-growing (*L*. *braziliensis*)." Lainson and Shaw (1972): First proposal of complexes of species for Neotropical *Leishmania* causing CL: the mexicana complex and the braziliensis complex. Schnur and colleagues (1972): serotype *Leishmania* with promastigotes excreted factors. Ranquein (1973): First proposal of a separate genus for *Sauroleishmania*. Bray (1973): Use the systematic concept for description of *Leishmania* species. Vickerman (1976): Proposed *Leishmania* that do not infect mammals as “not strictly being” *Leishmania* species, giving the status "Incertae sedis" to *Leishmania* isolated from reptiles. Gardener (1977): Proposed a taxonomy of the *Leishmania* genus that includes nomenclature, classification, and synonomies for the principal species and a list of species that do not normally infect humans. Hommel (1978), Wilson and Southgate (1979): Consider the identification and nomenclature under two titles of “traditional” and “modern” taxonomic criteria. Consider parasites that do not infect mammals as “not strictly being” *Leishmania* species. Lainson and Shaw (1979): Proposed a revised classification for American *Leishmania* species, based on their developmental patterns in *Lutzomyia longipalpis*. Subdivision into three groups: (i) Hypopylaria (*L*. *agamae* and and *L*. *ceramodactyli*), (ii) Peripylaria (*L*. *braziliensis* complex), (iii) Suprapylaria (*L*. *donovani*, *L*. *mexicana*, *L*. *hertigi*, and *L*. *tropica* complexes). Tait (1980): Suggests sexual recombination in trypanosomatids. Saf'janova (1982): Created a subgenus of *Leishmania* and proposed the term of *Sauroleishmania* Ranque, 1973 for parasites infecting lizards. Le Blancq and Peters (1986): Consider isoenzyme electrophoresis as a discriminatory system for *Leishmania* identification. Lainson and Shaw (1987): Division of *Leishmania* genus into two subgenera, based on the developmental pattern of *Leishmania* in the sand fly's gut: *Leishmania* (Suprapylorian) and *Viannia* (Peripylorian). Rioux and colleagues (1990): New classification of the *Leishmania* genus based on the use of intrinsic and extrinsic characters with Linnean and Adansonian methods. WHO (1990): Categorised the *Leishmania* species into three subgenera: *Leishmania*, *Sauroleishmania*, and *Viannia*. Momen (1993): Proposes the synonimy of *L*. *chagasi* (responsible for VL in the New World) and *L*. *infantum*. Shaw (1994): Proposes that the genus *Leishmania* encompass 30 species infecting mammals and 21 species infecting human. Cupolillo and colleagues (1994): Describe the monophyly of the subgenus *Viannia*. Dedet and colleagues (1999): Categorize the history of *Leishmania* classification into four periods of Linnean classifications, Adansonian classifications, phenetic classifications, and phylogenetic classifications.
2000 until now	Cupolillo and colleagues (2000), Schoenian and colleagues (2010): *Leishmania* genus composed of two groups: (i) *Euleishmania* (*Leishmania* and *Viannia* subgenera) and (ii) *Paraleishmania* (*L*. *hertigi*, *L*. *deanei*, *L*. *colombiensis*, *L*. *equatorensis*, *L*. *herreri*, and *Endotrypanum* species). Moreira and colleagues (2004): Present an updated classification of kinetoplastid protists. Fraga and colleagues (2010): New concepts, based on molecular data, concerning the reduction of the number of species, suppression of some species, and downgrading some to subspecies level. Kuhls and colleagues (2011), Leblois and colleagues (2011): Import of *L*. *infantum* (ca. 500 years ago) from the Old World (namely Portugal) to the New World as a result of finding a suitable vector there. Lukeš and colleagues (2014): Trypanosomatidae family consists of 13 genera: *Trypanosoma*, *Phytomonas*, *Leishmania*, *Leptomonas*, *Crithidia*, *Blastocrithidia*, *Herpetomonas*, *Sergeia*, *Wallacemonas*, *Blechomonas*, *Angomonas*, *Strigomonas*, and *Kentomonas*.

Comprehension of the evolutionary relationships between sandflies and *Leishmania* is crucial for the future prediction of *Leishmania* transmission patterns, leishmaniasis epidemiology, and for developing intervention and control strategies. To achieve such an understanding, better information on the worldwide distribution of *Leishmania* parasites in relation to their sandfly vectors and intermediate hosts will be required. It is therefore necessary to obtain information on the origin of *Leishmania* and phlebotomine sandflies and their chronological history of coevolution. In this paper, we present a detailed review of the relevant literature on the Phlebotominae and *Leishmania* and update and discuss theories on their classification, origin, evolution, and dispersion.

## Sandflies

Among more than 800 recognized sandfly species, approximately 464 species are found in the New World and 375 in the Old World [3,4]. The classification of both Old and New World sandflies has historically been based mainly on a phenetic approach to identifying overall similarity relationships between genera and subgenera, rather than on ancestor–descendant relationships. This approach has led to a proliferation of taxa, particularly at the subgeneric level, and to the simplification and incorporation of higher taxonomic categories into species. Sandflies belong to the order Diptera, suborder Nematocera, family Psychodidae, and subfamily Phlebotominae. Initially, studies on phlebotomine sandfly taxonomy were exclusively based on morphological aspects of dead specimens. Because of the introduction of several new methods, such as chromosome analysis, multivariate morphometrics, laboratory rearing and colonization, isoenzyme, molecular and phylogenetic analysis and, more recently, mass spectrometry, our knowledge of phlebotomine sandfly systematics has increased. These advances have led to better identification and classification of sandfly specimens, which together with an appreciation of sandfly flight range (approximately 1.5 km per day), have helped to clarify the intraspecific and interspecific variations within sandfly subgenera and populations. A large portion of the literature regarding phlebotomine sandfly systematics addresses their general classification and relationships with other groups [3,5–8] as well as the phylogenetics of the Psychodidae, based on insect fossils [9], phlebotomine sandfly evolution [5], phenetic and phylogenetic analyses of phlebotomine sandflies [10], and the molecular systematics and phylogenetic relationships of phlebotomines using DNA analysis [11]. Many classification systems for phlebotomine sandflies have been proposed since that of Newstead 1911, including those of Abonnenc, Davidson, Fairchild, Leng, Lewis, Quate, and Theodor. However, despite this extensive literature, there is no universal agreement regarding the ranking of taxa above the species level.

The history of sandfly taxonomy can be roughly divided into two distinct periods (Table 2). During the first period, taxa were distinguished according to the analysis of certain external structures (e.g., the structure of the male genitalia, wing venation indices and other external measurements, known as phlebotometry). In the second period, descriptions of internal structures such as the spermathecae, cibarium, and the pharynx were employed [12]. Based on the classification performed by Theodor [6,13], Lewis et al. [14] have proposed subdivision of the phlebotomine sandflies into two genera for Old World species, *Phlebotomus* (Rondani) and *Sergentomyia* (França), and three genera for New World species, *Lutzomyia* (França), *Brumptomyia* (França and Parrot), and *Warileya* (Hertig). The genus *Chinius* (Leng, 1987) belongs to a distinct taxon that is used for some Chinese sandfly species with primitive characters [15]. Rispail and Léger [10] proposed a new genus and subgenus classification for Old World sandflies, based on a morphological study suggesting their division into seven genera, including *Phlebotomus*, *Australophlebotomus*, *Idiophlebotomus*, *Spelaeophlebotomus*, *Sergentomyia*, *Spelaeomyia*, and *Chinius* (Table 2). In addition to the mentioned classification, some subgenera from the genus *Phlebotomus*, such as *Abonnencius* and *Legeromyia*, have been recently described and could be retained until a complete classification is proposed for the entire genus *Phlebotomus*.

**Table 2:** History of sandfly descriptions.

Century	Author (Year): Description
17th century	Bonanni (1691): First recognizable description of a sandfly as a species of *Culex*, or mosquito.
18th century	Linnaeus (1735): Description of Angioptera in the insect order that includes the Tipulary flies. Scopoli (1786): Description of *Phlebotomus papatasi* (*Bibio papataci*) as first species of described "Psychodidae," with no mention of a particular classification level. Latreille (1796): Description of the "*Pschoda*" genus that diverges from *Bibio* and *Tipula*.
first half of 19th century	Meigen (1818): Description of the Muchen (Tipularia) family that encompasses: Eulermuchen, Gallmucken (Gallicolae). Latreille (1825): Changed Tipulariae into Nemocera (Nematocera) family that included the tribe of Tipulariae and the group of Gallicolae (Psychode). Newman (1834): Gathered *Psychoda* genus in the order of Psychodite (Currently known as Psychodidae). Rondani (1840): Named sandflies as "Flebotomus" and put them into the tribe of Flebotomidae, family of Flebotominae. Renamed later as "Phlebotomus" by Lewis (1845). Rondani (1843): Includes sandflies in the tribe of Tipulidae, family of Hebotomina. Loew (1844): Description of *Haemasson minutus* (*Sergentomyia minuta*) that belongs to the family of "*Tipularia gallicola*," Psychodina. Walker (1848): Gathered *Psychoda* and *Sycorax* in the family of Tipularia, Noctuaeforme. Zetterstedt (1850): Includes *Psychoda* genus into the Psychodidae family.
second half of 19th century	Walker (1851): Considered the Phlebotomidae as a family belonging to Diptera. Bigot (1854), Rondani (1864), Schiner (1864): Gathered *Phloebotomus*, *Psychoda*, and some other genera in the Psychodidae family. Rondani (1856): Separation of the Phloebotomidae into Phloebotomina and Psychodina. Walker (1856): Gathered *Sycorax* and *Psychoda* and some other genera in the Phlebotomidae family. Loew (1862): Subdivided the Psychodidae family into Psychodina and Phlebotomina. Philippi (1865): Included the *Psychoda* genus into the "*Tipularia gallicola*" family. Hennig (1872): Proposed to use the name "Psychodites" as the generic name of fossil sandflies. Rondani (1873): Classification of sandflies into the Tipulidae tribe, family of Hebotomina (probably a syntax error). Eaton (1895), Kertesz (1902): Subdivided the Psychodidae into the Psychodinae and Phlebotominae subfamilies.
first half of 20th century	Kertesz (1903): Includes *Phlebotomus* and *Sycorax* into the Phlebotominae subfamily. Newstead (1911): First systematic study of the *Phlebotomus* genus. Subdivision of sandflies based on the dorsum hairs of the abdomen: erected or recumbent. Franca (1919, 1920): Subdivided sandfly species into *Phlebotomus* and *Prophlebotomus* subgenera. Formation of the first New World subgenus "*Lutzia*," encompassing *Phlebotomus longipalpis* Lutz and Neiva, 1912. Franca and Parrot (1921): Use phlebotometry to subdivide the *Phlebotomus* genus into five subgenera: *Phlebotomus*, *Prophlebotomus*, *Brumptomyia*, *Lutzia* (*Lutzomyia*), and *Sergentomyia*. Franca (1921): Proposed three subgenera; *Phlebotomus*, *Sergentomyia*, and *Lutzia*. Tonnoir (1922): Separated *Trichomyia* and *Sycorax* from the Phlebotominae and included them into the Trichomyiinae subfamily. France (1924): Substituted the name *Lutzia* for *Lutzomyia*. Adler and Theodor (1926): Highlighted the taxonomic value of the pharyngeal armatures and the spermathecae morphology. Sinton (1928): Noted a correlation between species defined by Newstead on the basis of erected or recumbent hairs and the female spermathecae morphology. Divided sandflies into three groups: erect-haired, recumbent-haired, and intermediate species. Dyar (1929): Updated the knowledge of the American flebotomíneos, proposing *Brumptomyia* (type species: *P*. *brumpti*), *Lutzomyia*, *Neophlebotomus* (type species: *P*. *malabaricus*), and *Shannonomyia* (type species: *P*. *panamensis*) subgenera. Adler and Theodor (1929): Defined sandflies as a formal member of the Phlebotomidae family. Nitzulescu (1931): Description of *Larroussius* and *Adlerius* subgenera, based on the pharyngeal armature and spermathecae structure. Proposed five subgenera: *Phlebotomus* s. str., *Larroussius* (type species: *P*. *major*), *Adlerius* (type species: *P*. *chinensis*), *Sintonius* (type species: *P*. *hospittii*), and *Brumptius* (type species: *P*. *minutus*). Sinton (1931): First illustrated identification keys for the Indian subcontinent sandflies. Theodor (1932): Phlebotominae subfamily composed of three tribes, further subdivided into genera and subgenera. Parrot (1934): *Phlebotomus* genus with two subgenera: *Phlebotomus* and *Prophlebotomus*. Raynal (1935): Tentative classification based on the spermathecae structure, male genitalia, and pharynx morphology. Mangabeira (1942): Created five subgenera for American sandfly species: *Evandromyia*, *Psychodopigus*, *Viannamyia*, *Pressatia*, and *Castromyia*. Dampf (1944): Put *Prophlebotomus* and *Brumplills* in synonymy with *Sergentomyia*, agreed with the subgenera *Brumptomyia*, *Shannonomyia*, *Castromyia*, and *Pintomyia*. Addis (1945): Created *Dampfomyia* as a new Neotropical subgenus. Kirk and Lewis (1946): Modified Parrot's (1934) classification and proposed three subgenera: *Phlebotomus*, *Sintonius*, and *Prophlebotomus*. Theodor (1948): Noted that two distinct periods characterize the progress in sandflies taxonomy: the first one that uses external morphological characters (phlebotometry) and the second one that uses internal characters. Four genera: *Phlebotomus* and *Sergentomyia* in the Old World, *Lutzomyia* and *Brumptomyia* in the New World. Description of six subgenera (*Paraphlebotomus*, *Synphlebotomus*, *Euphlebotomus*, *Anaphlebotomus*, *Australophlebotomus*, and *Spelaeophlebotomus*) that with three previously described (*Phlebotomus*, *Larroussius*, and *Adlerius*) made nine subgenera in total. Subdivided the *Sergentomyia* genus into three subgenera (*Sergentomyia*, *Sintonius*, and *Spelaeomyia*). Hertig (1948), Fairchild (1949): Description of *Warileya* (type species: *W*. *phlebotomanica*) and *Hertigia* (type species: *H*. *hertigi*) genera.
second half of 20th century	Jung (1954): Defines the Sycoracinae subfamily. Barretto (1955): Challenges Theodor's classification, proposed *Brumptomyia* and *Warileya* genera as being constitutive of New World species (166 species for the Old World and 199 from New World). Fairchild (1955): Subdivided Psychodidae into Phlebotominae (*Nemopalpus* and *Bruchomyia*), Trichomyiinae (*Horaiella* and *Sycorax* and others), and Psychodina. Theodor (1958): Erection of *Parrotomyia*, *Rondanomyia*, and *Grassomyia* as new subgenera of the *Sergentomyia* genus. Quate and Fairchild (1961): Addition of *Idiophlebotomus* as a new subgenus of the *Phlebotomus* genus. Barretto (1961): Stated that New and Old World sandflies must be phylogenetically distinct. Creation of the subgenus *Trichopygomyia* in the *Lutzomyia* genus. Barretto (1962): Confirmation of *Warileya*, *Brumptomyia*, and *Lutzomyia* genera in the New World and subdivision of *Lutzomyia* into fifteen subgenera: *Lutzomyia* s.str., *Pintomyia*, *Evandromyia*, *Psychodopygus*, *Viannamyia*, *Pressatia*, *Dampfomyia*, *Micropygomyia*, *Sciopemyia*, *Helcocyrtomyia*, *Trichophoromyia*, *Coromyia*, *Trichopygomyia*, *Nyssomyia*, and *Psathyromyia*. Theodor and Mesghali (1964): Erection of *Parvidens* as a new subgenus of *Sergentomyia*. Rohdendorf (1964): Included sandflies in the Phlebotomidae family. Separated sandflies from other Psychodidae because of their blood feeding habit. Theodor (1965): *Hertigia*, *Warileya*, *Brumptomyia*, and *Lutzomyia* genera for the New World. Subdivision of *Lutzomyia* into eight subgenera and 16 species groups. Perfil'ev (1966): Proposed a taxonomy based on external characters (phlebotometry) and internal structures (e.g., cibarium, pharynx, or spermathecae). Lewis (1971): Agrees with Perfil'ev (1966), divided Phlebotomidae into six genera (two in the Old World and four in the New World). Subdivided the *Phlebotomus* genus into 11 subgenera and *Sergentomyia* into six. Forattini (1971): Proposed seven genera for New World sandflies: *Brumptomyia*, *Lutzomyia*, *Pintomyia*, *Psychodopygus*, *Viannamyia*, *Pressatia*, and *Warileya*. Divides the *Lutzomyia* genus into six subgenera: *Lutzomyia*, *Dampfomyia*, *Micropygomyia*, *Coromyia*, *Trichopygomyia*, and *Barretomyia*. Hennig (1972): Considered Phlebotominae as a monophyletic group composed of three monophyletic genera: *Phlebotomus*, *Sergentomyia* (without *Parvidens*), and a genus gathering species from the *Brumptomyia* and *Lutzomyia* genera. Recognized the subfamilies Bruchomyiinae, Phlebotominae, Trichomyiinae, and Psychodinae within the Psychodidae family. Trichomyiinae familly encompasses three extinct genera (*Eophlebotomus*, *Eatonisca*, *Pasthon*) and three extant genera (*Horaiella*, *Sycorax*, *Trichomyia*). Abonnenc (1972): Agreed with Fairchild’s (1955) classification, recognised only three genera: *Phlebotomus*, *Warileya*, and *Hertigia*. Gathered *Spelaeophlebotomus* and *Idiophlebotomus* into the *Phlebotomus* genus. Raised the *Phlebotomus*, *Sergentomyia*, and *Lutzomyia* subgenera to a generic rank. Hennig (1973): Considered the Psychodoidae superfamily as a monophyletic infraorder of Psychodomorpha. Duckhouse (1973): Six subfamilies for the Psychodidae family: Phlebotominae, Bruchomyiinae, Sycoracinae, Trichomyiinae, Horaellinae, and Psychodinae. Forattini (1973): Considered *Phlebotomus*, *Sergentomyia*, and *Lutzomyia* as genera. Gathered the *Hertigia* genus within the Bruchomyiinae subfamily. Proposed ten genera for the Phlebotominae subfamily. Lewis (1973): included *Hertigia* (currently known as *Warileya*) into the Phlebotominae subfamily. Young and Fairchild (1974): Proposed a classification similar to Theodor (1965), with some modifications. Lewis (1974): Six genera for the Phlebotomidae subfamily (two for Old World species and four for the New World ones). Lewis (1975): 11 subgenera for *Phlebotomus* and six for *Sergentomyia*. Abonnenc and Leger (1976): The Phlebotomidae family with three subfamilies: Euphlebotominae (only Old World), Neophlebotominae (only New World), and Disphlebotominae (Old and New World). Lewis and colleagues (1977), Lewis (1978): First stable classification of Phlebotominae with five genera: *Warileya* (two subgenera), *Phlebotomus* (ten subgenera), *Sergentomyia* (seven subgenera with 54 unplaced species), *Brumptomyia*, and *Lutzomyia* (26 subgenera and 19 unplaced species). Ready and colleagues (1980): Proposed a “flexible” classification with “exclusive” characters supporting the proposed genera of *Phlebotomus*, *Sergentomyia*, *Brumptomyia*, *Warileya*, and *Psychodopygus*, without considering *Lutzomyia*. Lewis (1982): Described and added a new subgenus, *Kasaulius*. Published a distribution map for Old World sandflies. Artemiev and Neronov (1984): 14 genera for Phlebotominae: *Australophlebotomus*, *Brumptomyia*, *Demeillonius*, *Grassomyia*, *Hertigia*, *Idiophlebotomus*, *Lutzomyia*, *Parvidens*, *Phlebotomus*, *Psychodopygus*, *Sergentomyia*, *Spelaeomyia*, *Spelaeophlebotomus*, and *Warileya*. Description of the *Transphlebotomus* subgenus. Leng (1987): Description of new genus of *Chinius*. Artemiev (1991): Two tribes, seven subtribes, 24 genera, 40 subgenera, and 70 species constitute the Phlebotominae subfamily. Divided Old World sandflies into *Phlebotomus*, *Australophleotomus*, *Idiophlebotomus*, *Spelaeophlebotomus*, *Sergentomyia*, *Spelaeomyia*, *Chinius*, and *Parvidens*. Lane (1993): Genus *Phlebotomus* composed of 12 subgenera. Added the genus *Chinius* into the Phlebotominae subfamily. Young and Duncan (1994): Neotropical sandflies composed of *Lutzomyia*, *Brumptomyia*, and *Warileya*. Galati (1995): Created a new subtribe (Sergentomyiina) that gathered species from the *Sergentomyia* genus and some reptile-biting species from the *Lutzomyia* genus. Division of Phlebotominae into Hertigiini (Hertigiina, Idiophlebotomina) and Phlebotomini (Phlebotomina, Australophlebotomina, Brumptomyiina, Sergentomyiina, Lutzomyiina, and Psychodopygina) tribes. Rispail and Leger (1998): Proposed seven genera for Phlebotominae sandflies: *Phlebotomus*, *Australophlebotomus*, *Idiophlebotomus*, *Spelaeophlebotomus*, *Sergentomyia*, *Spelaeomyia*, and *Chinius*. The *Phlebotomus* genus includes nine subgenera: *Adlerius*, *Anaphlebotomus*, *Euphlebotomus*, *Kasaulius*, *Larroussius*, *Paraphlebotomus*, *Phlebotomus*, *Synphlebotomus*, and *Transphlebotomus*. The *Sergentomyia* genus includes six subgenera: *Demeillonius*, *Grassomyia*, *Neophlebotomus*, *Parrotomyia*, *Sergentomyia*, and *Sintonius*.
2000 until now	Galati (2003): Proposed to subdivide the Phlebotominae familly into two tribes: Hertigiini (subtribes of Hertigiina and Idiophlebotomina) and Phlebotomini (subtribes of Phlebotomina, Australophlebotomina, Brumptomyiina, Sergentomyiina, Lutzomyiina, and Psychodopygina). Galati (2009): Upgraded many of the *Lutzomyia* subgenera, cited in Young and Duncan, 1994, to a generic status. Galati (2014): Revised the classification proposed by Galati, 2003, leading to an increase in genera numbers.

A classification first proposed by Lewis et al. [14] and later reviewed by Young and Duncan [8] subdivides the Neotropical sandflies into *Lutzomyia*, *Brumptomyia*, and *Warileya*. This classification is still accepted by a majority of sandfly taxonomists. A new system of classification has been proposed by Galati [3], who revised the existing proposals for New World sandflies. The system recognized 464 species of Neotropical phlebotomine sandflies, grouped into 23 genera, 20 subgenera, three species groups, and 28 series. This classification includes a complete review and reorganization of the subfamily Phlebotominae, which is further classified into two tribes, Hertigiini (Hertigiina and Idiophlebotomina subtribes) and Phlebotomini (Phlebotomina, Australophlebotomina, Brumptomyiina, Sergentomyiina, Lutzomyiina, and Psychodopygina subtribes).

In 2014, Galati revised her previous publication and proposed a new version of classification for Phlebotominae sandflies [3,16]. Based on her classification, the Phlebotomini tribe includes 931 extant species (916 valid species and 15 with uncertain taxonomic status) classified in six subtribes:

- Phlebotomina (*Phlebotomus* genus, 110 spp.)
- Australophlebotomina (*Australophlebotomus* genus, ten spp.)
- Brumptomyiina (*Brumptomyia* [26 spp.] and *Oligodontomyia* [three spp.] genera)
- Sergentomyiina (*Sergentomyia* [310 spp.], *Deanemyia* [five spp.], and *Micropygomyia* [55 spp.] genera)
- Lutzomyiina (*Sciopemyia* [eight spp.], *Lutzomyia* [74 spp.], *Migonemyia* [seven spp.], *Pintomyia* [57 spp.], *Dampfomyia* [20 spp.], *Expapillata* [two spp.], *Pressatia* [eight spp.], *Trichopygomyia* [16 spp.], and *Evandromyia* [42 spp.] genera)
- Psychodopygina (*Psathyromyia* [43 spp.], *Viannamyia* [four spp.], *Martinsmyia* [11 spp.], *Bichromomyia* [six spp.], *Psychodopygus* [40 spp.], *Nyssomyia* [20 spp.], and *Trichophoromyia* [39 spp.] genera).

The genus *Edentomyia*, including one species (*Edentomyia piauiensis*), was described by Galati [3] without indicating the taxonomic position in the Phlebotomini tribe.

The Hertigiini tribe contained two subtribes of Hertigiina (*Warileya* and *Hertigia* genera) and Idiophlebotomina (*Spelaeophlebotomus*, *Idiophlebotomus*, and *Chinius* genera), with five genera and 28 extant species.

**Fig 1:**
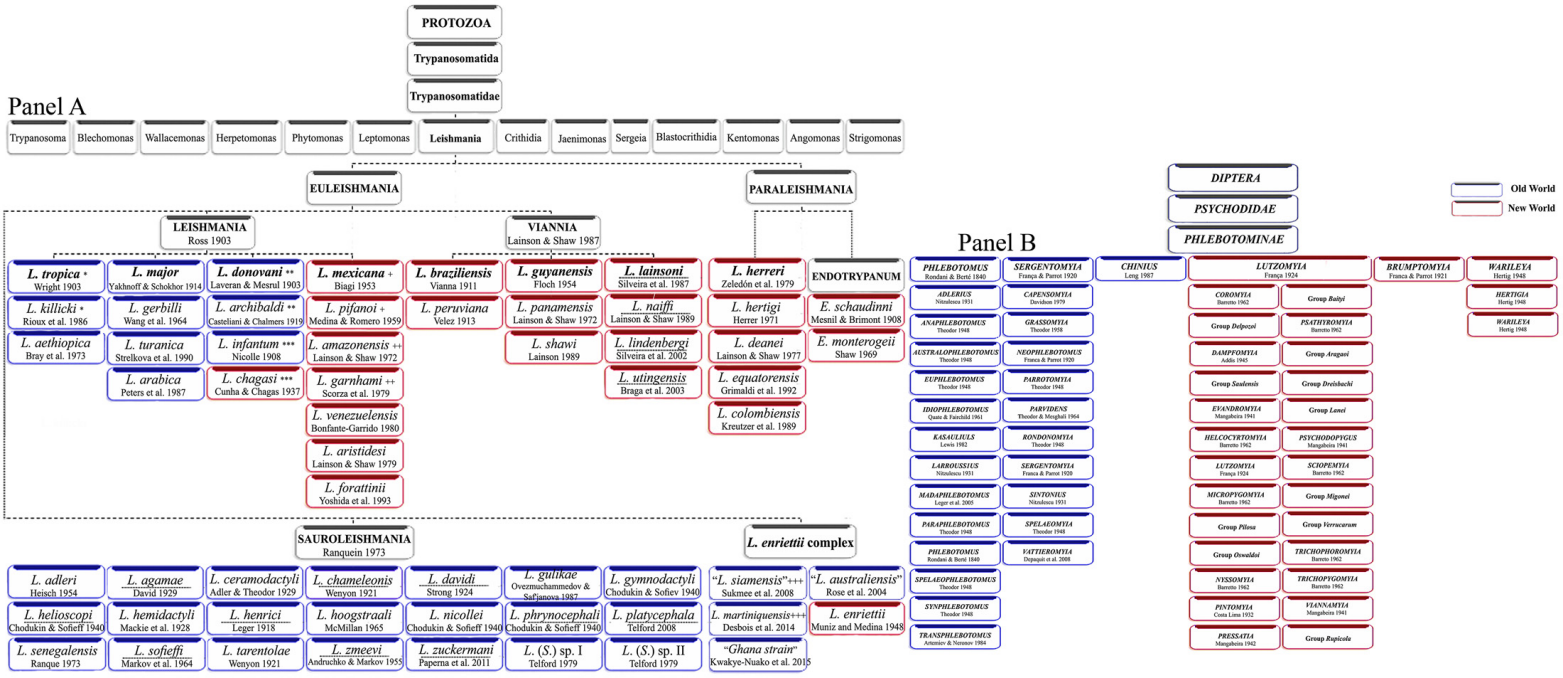
Updated classification of *Leishmania* and sandfly. **Panel A**. Classification of *Leishmania* species. **Panel B**. Phlebotominae sandfly classification, according to Theodor [6,13], Quate and Fairchild [163], Theodor and Mesghali [22], Lewis [5], Leng [15], and Young and Duncan [8].

Currently, a conservative approach based on practical criteria has led to subdivision of the Phlebotominae into six genera: three genera from the Old World (*Phlebotomus* [13 subgenera], *Sergentomyia* [ten subgenera], and *Chinius* [four species]) and three from the New World (*Lutzomyia* [26 subgenera and groups], *Brumptomyia* [24 species], and *Warileya* [six species]) (Fig 1) [8,17]. This classification is currently widely used.

## Old World Sandflies

The Old World sandflies include three genera: *Phlebotomus*, *Sergentomyia*, and *Chinius*, which are found in the Palaearctic, Afrotropical, Malagasy, Oriental, and Australian regions.

Genus *Phlebotomus* (Rondani and Berté, 1840) includes 13 subgenera: *Adlerius*, *Anaphlebotomus*, *Australophlebotomus*, *Euphlebotomus*, *Idiophlebotomus*, *Kasauliuls*, *Larroussius*, *Madaphlebotomus*, *Paraphlebotomus*, *Phlebotomus*, *Spelaeophlebotomus*, *Synphlebotomus*, and *Transphlebotomus* (Fig 1). They are present only in the Old World and are particularly prevalent in the Palaearctic region, which is the main temperate area of the Old World. Most *Phlebotomus* species are inhabitants of semiarid and savannah areas rather than forests. Therefore, the geographical distribution of the genus *Phlebotomus* extends from the Mediterranean, Afrotropical, Middle East, and Oriental regions to central Asia. They are found in a wide range of altitudes, from Jericho of Palestine (~300 metres below sea level) to Mashad in Iran (3,600 metres above sea level). In tropical areas, only a few species of *Phlebotomus* are present, such as in sub-Saharan Africa, Southeast Asia, or the Pacific region. They feed mainly on mammals, although there are some exceptions. This genus includes many human blood feeders and some endophilic species. All of the vectors of human cutaneous and visceral leishmaniasis found in Eurasia and Africa belong to this genus.

Genus *Sergentomyia* (Franca and Parrot, 1920) is subdivided into ten subgenera: *Capensomyia*, *Grassomyia*, *Neophlebotomus*, *Parrotomyia*, *Parvidens*, *Rondonomyia*, *Sergentomyia*, *Sintonius*, *Spelaeomyia*, and *Vattieromyia* (Fig 1). This genus contains some ungrouped species. Members of this genus are widespread in the Old World and are dominant in tropical areas where *Phlebotomus* species are scarce. Their distribution comprises Afrotropical, Oriental, and Australasian regions, the Indian subregion, sub-Saharan Africa, and Asia. Most species are likely to feed chiefly on cold-blooded vertebrates, but some species occasionally bite mammals [18]. Some *Sergentomyia* specimens have been found to contain *Sauroleishmania* (a subgenus of *Leishmania*) and *Trypanosoma* parasites that are often identified as parasites from lizards [19], but current evidence indicates human *Leishmania* parasites are not transmissible by *Sergentomyia* flies [20].

Genus *Chinius* (Leng, 1987) includes four known species: *Chinius junlianensis*, *C*. *barbazani*, *C*. *eunicegalatiae*, and *C*. *samarensis*. The geographical repartitioning of *Chinius* corresponds to the classical Oriento-Australasian track, and they are found in caves in high mountainous regions.

The geographical distribution of the currently known Old World sandfly species encompasses the following areas:

1. The Palaearctic region: species belonging to the *Phlebotomus* genus are dominant in the Palaearctic region, as it is the main temperate area of the Old World. Nearly 200 sandfly species belong to various *Phlebotomus* subgenera; *Adlerius*, *Anaphlebotomus*, *Euphlebotomus*, *Idiophlebotomus*, *Larroussius*, *Paraphlebotomus*, *Phlebotomus*, *Synphlebotomus*, and *Transphlebotomus*, as well as the *Chinius* and *Sergentomyia* genera, are found in the Palaearctic region. (Iran [6,21,22], Pakistan [23], the former U.S.S.R. [12], France [24], Turkey [25], Morocco [26], Yemen [27], Spain [28], Tunisia [29], Afghanistan [30], Saudi Arabia [31], Iraq [32], Algeria [33], Egypt [34], Greece [35], China [15,40], Jordan [4,10,36–39].)
2. The Afrotropical region: subgenera of *Anaphlebotomus*, *Larroussius*, *Paraphlebotomus*, *Phlebotomus*, *Spelaeophlebotomus*, and *Synphlebotomus* from the genus *Phlebotomus*, together with the genus *Sergentomyia*, are distributed in this region. Surprisingly, however, some *Phlebotomus* species that are known to be inhabitants of this region are absent from western Afrotropical regions. (Gabon [41], Sudan [17], Central African Republic [4,10,39,42], Ethiopia [43], Southern Africa [44].)
3. The Malagasy region (Madagascar and nearby Indian Ocean islands): Species belonging to the genera of *Phlebotomus* (*Anaphlebotomus* and *Madaphlebotomus* subgenera) and *Sergentomyia* are present in this region. Despite their presence, no sandfly species has been reported as a disease vector in this region [45].
4. The Oriental region: Approximately 122 sandfly species belonging to the *Phlebotomus*, *Chinius*, and *Sergentomyia* genera are present in this region. In the mainly dry western area, the sandfly fauna is essentially Eremian (The Eremian zone has an arid climate, and its vegetation ranges from barely vegetated desert and hills to a variety of semiarid shrub savannas, semiarid tussock grasslands, and hummock grasslands). In eastern India, *Phlebotomus argentipes* is an important vector of kala azar. In the far eastern area, including Vietnam, sandflies known to bite humans are rare or absent, and there appear to be rather few phlebotomine species in this area, with the exception of the Philippines [46,47,48].
5. The Australian region: the Australasian phlebotomine fauna is bipolar in origin, with the genus *Phlebotomus* (*Australophlebotomus*: eight spp.) originating from the south and the subgenus *Idiophlebotomus* (one sp.) and *Sergentomyia* (24 spp.) from the north [49]. The co-occurrence of some sandfly species (e.g., *S*. *hoogstraali*, *S*. *vanella*) in both Australia and New Guinea supports the hypothesis proposed by Schodde and Calaby [50] regarding the simultaneous development of the New Guinea sandfly fauna along with the eastern Australia sandflies. Sandflies are generally abundant in both regions where there is rainfall of less than 635 mm, as well as in the wetter northern zone, where the dry season is long. These areas, unlike the Eremian zone of the northern hemisphere, support only a few *Phlebotomus* species, and humans and livestock are rarely attacked (New Guinea [49,51–53]).

## New World Sandflies

The New World sandflies include three genera: *Lutzomyia*, *Warileya*, and *Brumptomyia*, which are found in the Nearctic and Neotropical regions:

Genus *Lutzomyia* Franca, 1924. This is a large genus, with nearly 434 species and several subgenera, including the *Coromyia* (*Delpozoi* group), *Dampfomyia* (*Saulensis* group), *Evandromyia*, *Helcocyrtomyia*, *Lutzomyia*, *Micropygomyia* (*Pilosa* and *Oswaldoi* groups), *Nyssomyia*, *Pintomyia*, *Pressatia* (*Baityi* group), *Psathyromyia* (*Aragaoi*, *Dreisbachi*, and *Lanei* groups), *Psychodopygus*, *Sciopemyia* (*Migonei* and *Verrucarum* groups), *Trichophoromyia*, *Trichopygomyia*, and *Viannamyia* (*Rupicola* group), as well as some ungrouped species (Fig 1). The *Lutzomyia* genus is more diverse than its Old World counterparts. Nevertheless, vector species are found only in some subgenera (*Nyssomyia*, *Psychodopygus*, and *Lutzomyia* s.str.). Sandflies are of little importance in temperate North America but are abundant in tropical America. *Lutzomyia* is the most important genus in terms of species diversity and medical importance and exhibits a wide dispersion area. Species of this genus are found only in the New World, with a distribution ranging from the southern areas of the Nearctic region throughout the Neotropical ecozone. Sandflies are found mainly in forest areas in Central and South America. Wide morphological variations have been described for *Lutzomyia* species, which are greater than those of the Old World species. Therefore, the classification of *Lutzomyia* species remains largely unresolved and relies on divisions based on morphological taxonomic characters that are still controversial.

Genus *Warileya* (Hertig, 1948) includes six species, which are mainly found in the Neotropical ecozone.

Genus *Brumptomyia* (Franca and Parrot, 1921) comprises approximately 24 species, which are broadly distributed in Central and South America. None of these species are known to bite humans. *Brumptomyia* species constitute a group of sandflies commonly associated with armadillo burrows and sometimes tree trunks. The specific identification of species belonging to this genus is based entirely on male structures [3,54,55].

Sandflies from the New World are present only in Nearctic and Neotropical ecozones:

1. The Nearctic region: only 14 species, a majority of which come from the *Micropygomyia* subgenus, are present in the Nearctic, but five are restricted to this ecozone. Most of these species exhibit a preference for hot temperatures and humidity. The temperate climate found in the Nearctic is unfavourable for phlebotomine development, particularly for immature stages. This characteristic supports the idea that phlebotomine sandflies might have originated in the tropics, with only a few species dispersing into temperate regions. The sandfly species that are currently found in North America likely arose from the Palaearctic or from South America during the arid phase in the Tertiary period. Therefore, their decreased presence may be a consequence of the constant climatic fluctuations that have occurred during the Quaternary period, causing many sandfly species to become extinct or displaced into the tropics, where hotter and more humid conditions are present [3,56,57].
2. The Neotropical region: approximately 450 sandfly species are found in this ecozone. The distribution centre of the present-day *Lutzomyia* genus in the Neotropics is thought to be the forested lowlands present in the east of the Andes. This situation is probably a consequence of the dry periods that occurred during the Pleistocene that isolated conspecific populations, some of which became reproductively isolated and have colonized more humid areas present in the northern and western parts of the subcontinent [10]. The varied sandfly fauna present in wet areas includes many potential sandflies that feed on the blood of human beings. However, only a few are endophilic species (Colombia [58], Ecuador [59], Costa Rica [60], Peru [61], Brazil [62], French Guiana [63], Venezuela [3,8,55,64–67]).

## Sandfly Fossil Evidence

Fossils, including the remains of living organisms from the past, are one of the best forms of evolutionary evidence. They allow for comparisons with current organisms and are of particular importance in allowing knowledge of primitive character states (plesiomorphic) and derived specialized states (apomorphic) to be obtained. Fossils provide information about the origin of vector flies in relation to infectious agents, host coevolution, and geographic locations. Therefore, research on sandfly fossils is of great importance for highlighting the evolution and phylogeny of these insects. As mentioned above, phlebotomine sandflies are found in a wide range of ecozones, which could be due to their long evolutionary history with their origins in the Palaeozoic or Mesozoic eras [68].

Arthropods first arose towards the end of the Precambrian period, approximately 550 million years ago (MYA). The first Parainsecta appeared in the Devonian (408 MYA), and the earliest insect orders emerged during the subsequent Carboniferous period. Variegation continued to occur in the Permian (286 MYA), which was the period during which the Diptera arose. Psychodidae emerged later, during either the Jurassic [69] or the Triassic period [70]. This group was likely well diversified by the Cretaceous, and the majority of these species were likely to have been blood feeders. These observations together support the theory of a hypothetical phlebotomine-like ancestor for Psychodidae [9]. The sandflies most likely emerged during the Carboniferous and, thus, before the mammalian hosts of *Leishmania*. A common ancestor for Phlebotominae is thought to have occurred in the Triassic period (248 MYA) (Table 3).

**Table 3:** Evolution history of *Leishmania*, sandfly, and reservoir over the time along the geographical evolution of the Earth.

Geographical time (MYA: Million Years Ago)	PALEOZOIC							MESOZOIC			CAENOZOIC						
	PRECAMBRIAN (>544)	CAMBRIAN (544–505)	ORDOVICIAN (505–440)	SILURIAN (440–410)	DEVONIAN (410–360)	CARBONIFEROUS (360–286)	PERMIAN (286–245)	TRIASSIC (245–208)	JURASSIC (208–146)	CRETACEOUS (146–65)	PALAEOCENE (65–55)	EOCENE (55–38)	OLIGOCENE (38–25)	MIOCENE (25–5)	PLIOCENE (5–2.5)	PLEISTOCENE (2.5MYA-12TYA)	HOLOCENE (12TYA until now)
**GEOGRAPHICAL EVENTS**	Emergence of Atlas Mountains			Melting of the large glacial formations Emergence of the Land Plants			Continents joined (Pangea)	Seperation of the continents (235)	Separation of Gondwana from Pangea (180) Separation of Laurasia from Gondwana (180) Formation of Andes Mountains (200)	Separation of Africa and South America Formation of Bering straits Emergence of Rocky Mountains (70)	Formation of McKinley (Denali) Mountains (56)	Histricomorpha of Neotropics Emergence of Alps Mountains (50) Emergence of Himalayas Mountains (40)	Separation of Africa and Saudi Arabia plate Breaking the Bering land bridge	Cooling of the North Pacific	Formation of Panamamian Isthmus and physical unification between Nearctic and Neotropic	GlaciationsCooling and drying the earth (1.5–2.5) Emergence of Kilimanjaro mountain (750 TYA)	Warming trend of the earth (600–900)
***LEISHMANIA* sp.**	Emergence of Protozoa (750)		Emergence of eukaryote supergroup Excavata Appearence of the descendant of *Leishmania*		Emergence of the first digenetic protozoa, ancestor of other *Trypanosoma*, not *Leishmania*	Emergence of Trypanasomes (300)	Division of Trypanosomatidae following the evolution of Diptera (vector of *Leishmania*)		First digenetic protozoa, a possible ancestor of *Leishmania*	First *Leishmania* decendent in a reptile host, Evolving of *Sauroleishmania* from other genera of *Leishmania* First fossil of the genus *Leishmania* (*Paleoleishmania proterus*) (Burmese amber) (100) Divergence of Old World and New World *Leishmania*(90) Dixenous life cycle of *Leishmania* (85)	Distribution of *Leishmania* species after rodents’ emergence during Paleocene (after emergence of primitive mammals)	Predecessor of *L*. *donovani* group and *L*. *major* evolved from South America (36–46) Complete life cycle of *Leishmaia* (50)		*Leishmania* migration from Palearctic to Nearctic or inversly based on the hypotheses of Palearctic or Nearctic origin of *Leishmania* Adaptation of *Sauroleishmania* to the lizards *Paleoleishmania neotropicum* (Dominican amber) (20) Diverging the ancestor of *L*. *donovani* from other *Leishmania* species (14–24)	Dispersion of *Leishmania* into or out of the Neotropic region throught the Panamamian Isthmus	Divergence of *L*. *donovani* from *L*. *infantum* (1) Origination of *L*. *chagasi* from *L*. *infantum* in South America (500)	
**SANDFLY**	Emergence of the first Arthropodes (550)	Dispensation of the Arthropodes			Emergence of Parainsecta (408)	Emergence of Insects (360) First winged insect (300)	Emergence of Diptera (286) Earliest Psychodids	Emergence of Phlebotominae, common ancestor of the Old and New World sandfliesDifferentiation of the tribes Hertigiini and Phlebotomini	Emergence of Psychodids First record of the presence of the true sandflies (180) Separation of Old World and New World sandflies (200)	First hematophagus winged insect, ancestor of *Phlebotomus*, *Sergentomyia*, *Lutzomyia* (140) *Phlebotomites longifilis*, *P*. *brevifilis*, *Mesophlebotomites hennigi*, and *Libanophlebotomus lutfallahi*, fossil records from Lebanon amber (120) *Palaeomyia burmitis* (Burmese amber) (100) Emegence of the ancestor of *Phlebotomus* and *Sergentomyia* in the Palaearctic region		Emergence of the genus *Phlebotomus* *Phlebotomiella*/*Sergentomyia succini* (Baltic amber)	Separation of *Lutzomyia* and *Phlebotomus* genera	*P*. *(Phlebotomiella) tipuliformis* (Baltic amber) (20) *Lutzomyia (Helcocyrtomyia) paterna* (Mexican amber) (20) *Lutzomyia adiketis* (Dominican amber) (20) *Pintomyia falcaorum* (Dominican amber) (20) *Phlebotomus pungens* (Jordanian amber)			
**RESERVOIR**	Emergence of Animalia kingdom (700)						Emergence of Reptiles (285)	Emergence of Mammals (210)	Emergence of Lizards	Emergence of Marsupials (Opossums) Spread of *Leishmania* into the Neartic by primitive mammals through the Bering Strait	Placental mammals Primates (60) Rodents (55) Xenarthrans (55)	Emergence of the sloths	Emergence of Rodents (25) Emergence of Canides (dogs) Emergence of Caviomorph rodents (25)	Spread of the *Leishmania* from Palearctic to the New World, probably by an infected rodent Sigmodontinae (Cricetids) (20)	Emergence of Human being, genus *Homo* (3)		*Homo sapiens* (200 TYA)

To date, sixteen fossils representative of New World species have been described (15 from Dominican and one from Mexican amber). These fossils correspond to the *Lutzomyia* genus, including subgenera of *Lutzomyia* (one sp.), *Micropygomyia* (two spp.), *Pintomyia* (12 spp.), and *Psathyromyia* (one sp.) [71]. Additionally, some old amberic records of phlebotomine-like species have been recorded from the Old World, including some fossils deposited in France [72], Germany [73], Spain [74], Burma [75], and Lebanon [76], although the taxonomic placement of some of these species into the Phlebotominae is still unclear. The oldest known species of Phlebotominae are *Phlebotomites longifilis* (Hennig, 1972), *P*. *brevifilis (*Hennig, 1972), *Mesophlebotomites hennigi* (Azar, Solignac, Paicheler, and Bouchet, 1999), and *Libanophlebotomus lutfallahi* (Azar, Solignac, Paicheler, and Bouchet, 1999), for which there are fossil records described from Lebanon, in the south of the Tethys Sea, dated to approximately 120 MYA [5,9]. Since that time, the evolution of the Phlebotominae was likely to have been driven by major tectonic events and related climatic changes that affected the break up of Pangaea. Prior to 120 MYA, the Phlebotominae had likely remained on Pangaea for quite some time, from which separated sandfly faunas could have developed in the Old World and New World [5]. Sandfly fossil records as well as data on systematics strongly indicate that the current genera existed quite some time before the Mesozoic, 250 MYA [73]. *Palaeomyia burmitis* was found in Burmese amber dated from the Cretaceous period (100 MYA). Trypanosomatids associated with a fungal food source were discovered in the alimentary tract of sandfly larva. Another sandfly fossil, *P*. *(Phlebotomiella) tipuliformis* (Meunier, l905), was found in Baltic amber dated from the Eocene (20 MYA). This species may have lived in the forest and fed on thin-skinned reptiles [9,77]. *Sergentomyia succini* (Stuckenberg, 1975), is another sandfly fossil found in Baltic amber [77]. Additionally, *Phlebotomus pungens* (Loew, 1845), and *P*. *khludae* (Kaddumi, 2005) [78], reported from the Old World, both were discovered in Jordanian fossil amber. Sandflies from Mexican ambers from Chiapas were identified as *Micropygomyia patterna* (= *Lutzomyia paterna* [Quate, 1963]) and dated to the Miocene (20 MYA). This species is the first known phlebotomine among the current reptile-feeding species to exhibit narrow wings and to feed on blood [9,79]. A sandfly fossil found in Dominican amber was identified as a female of *Lutzomyia adiketis* and was dated to approximately 20 MYA. This discovery supports the hypothesis of the radiation of *Lutzomyia* species throughout the Neotropics. In addition to *Lutzomyia adiketis*, *Pintomyia falcaorum*, *Trichopygomyia killickorum*, *L*. *filipalpis*, *L*. *succini*, *L*. *miocena*, *L*. *paleopestis*, *L*. *schleei*, *P*. *brazilorum*, *P*. *paleotownsendi*, *P*. *paleotrichia*, and *M*. *brandaoi* were also found in this Miocene Dominican amber. Two other groups of fossils were found by Young and Lawyer [56] and Antoine et al. [80] in Dominican (14 specimens) and Peruvian (one specimen) ambers, dating from the Miocene. These specimens were not described by the authors that discovered the ambers.

Currently, there are two hypotheses that attempt to explain how the worldwide dispersion of sandfly ancestors occurred. The first hypothesis assumes that sandflies evolved in the Palaearctic ecozone during the Cretaceous period and were then isolated because of the breakup of Pangaea and underwent independent evolution, resulting in two subgenera, *Phlebotomus* (that has evolved during the Eocene) and *Lutzomyia* (which evolved during the Oligocene, after the breaking of the Bering bridge). These two genera include species that are involved in the transmission of *Leishmania* in the Old and New Worlds, respectively [81,82]. According to the second hypothesis, the similarities between the current sandfly taxa and those recorded in fossils, as well as their external positions on phenetic or cladistic trees, support the hypothesis that they existed in Gondwana before the continental separation [83].

## Leishmania

The Trypanosomatidae family consists of three dixenous genera (life cycle in vertebrates or plants and invertebrates)—*Trypanosoma*, *Phytomonas*, and *Leishmania*—11 monoxenous genera (life cycle in invertebrates only)—*Leptomonas*, *Crithidia* (together with *Leishmania* form the subfamily Leishmaniinae), *Blastocrithidia*, *Herpetomonas*, *Sergeia*, *Wallacemonas*, *Blechomonas*, and *Jaenimonas*—and three genera that are characterized by the presence of endosymbiotic bacteria and form the subfamily Strigomonadinae: *Angomonas*, *Strigomonas*, and *Kentomonas* [84–88].

*Leishmania* parasites belong to the Kingdom Protista (Haeckel, 1866), Class Kinetoplastea (Honigberg, 1963 emend. Vickerman, 1976), Subclass Metakinetoplastina (Vickerman, 2004), Order Trypanosomatida (Kent, 1880), Family Trypanosomatidae (Döflein, 1901), Subfamily Leishmaniinae (Maslov and Lukeš 2012), and Genus *Leishmania* (Ross, 1903).

*Leishmania* species are heteroxenous, meaning that they are able to colonize two hosts. They live in the phagocytes of the reticulo-endothelial system of mammals and in the intestinal tract of phlebotomine sandflies, although *Forcipomyia* spp. (Diptera: Ceratopogonidae) as well as some tick species have been reported as the potential vectors of *Leishmania* sp. [89–91]. Mammalian *Leishmania* species exhibit a worldwide distribution (Table 4). They are present in tropical and subtropical areas, including North, Central, and South America, as well as in the Mediterranean basin, Southeast Europe, the Middle East, Central and Southeast Asia, the Indian subcontinent, Africa, and recent reports also demonstrate their presence in Australia (Table 4). In the Malagasy region, with the exception of one case of canine leishmaniasis reported by Buck et al. [92], no autochthonous case of leishmaniasis has been reported. Alvar et al. [2] presented an overview of the occurrence of leishmaniasis and causative species in all affected countries. In the Old World, most *Leishmania* transmissions occur peridomestically in semiarid areas modified by humans, whereas New World parasites are often associated with sylvatic habitats, though some species exhibit predominantly peridomestic transmission. Host preference is also a major factor that affects the modality of *Leishmania* transmission by a vector that can occur among wild animals, from animals to man, or among people. Although predominantly gut-dwelling, *Leishmania* parasites were rarely detected also in salivary glands of sand flies. The presence of parasites in the glands was correlated with heavy infections of metacyclic promastigotes in the stomodaeal valve and thoracic midgut of the fly. Therefore, there was a strong correlation between infected glands and the intensity of infection in the midgut, linked to the presence of numerous metacyclic forms [93].

**Table 4:** Different *Leishmania* species of Old and New World, their synonymies, distributions, reservoirs, and their potential or proven vectors.

*Leishmania sp*.(synonymes)			Old and/or New World	Clinical Disease	Reservoir				Sandfly Vector (potential or proven)	Distribution	References
					Mammal	Human	Lizard	Insect			
***EULEISHMANIA***	***LEISHMANIA*** (growth in the midgut and foregut of sandfly)	*L*. *aethiopica*	OW	CL, DCL	X	X			*P*. *(Lar*.) longipes*, *P*. *(Lar*.) pedifer*, *P*. *(Par*.) sergenti*	East Africa (Ethiopia, Kenya)	[172]
		*L*. *amazonensis* (syn. of *L*. *garnhami*)	NW	CL, DCL, MCL	X	X			*Lu*. *(Lu*.*) diabolica*, *Lu*. *(N*.) flaviscutellata*, *Lu*. *(Lu*.) longipalpis*, *Lu*. *(Lu*.) nuneztovari anglesi*, *Lu*. *(N*.) olmeca novica*, *Lu*. *(N*.) olmeca reducta*, *Lu*. *(V*.*) townsendi*, *Lu*. *(N*.*) ylephiletor*, *Lu*. *(V*.*) youngi*	South America (Bolivia, Brazil, Venezuela)	[173,174]
		*L*. *arabica*	OW	_	X				*P*. *(P*.*) papatasi*	Saudi Arabia	[175]
		*L*. *aristidesi*	NW	_	X^R^				*Lu*. *(N*.*) olmeca bicolor*, *Lu*. *(N*.*) trapidoi*	Panama	[176]
		*L*. *donovani* (syn. of *L*. *archibaldi*)	OW	VL, PKDL	X	X			*P*. *(Pa*.) alexandri*, *P*. *(Eu*.) argentipes*, *P*. *(Syn*.) celiae*, *P*. *(Ad*.*) chinensis*, *P*. *(Ad*.*) longiductus*, *P*. *(Syn*.) martini*, *P*. *(La*.) orientalis*, *P*. *(Ad*.*) sichuanensis*, *P*. *(Sy*.*) vansomerenae*	Central Africa, South Asia, Middle East, India, China	[4,40,177]
		*L*. *gerbilli*	OW	_	X				*P*. *(P*.*) papatasi*	Central Asia, South Mongolia, Iran	[178]
		*L*. *forattinii*	NW	_	X^R^				*Lu*. *(Lu*.*) gasparviannai*	Brazil	[179]
		*L*. *infantum* (syn. of *L*. *chagasi*)	OW, NW	VL, CL	X	X			*P*. *(Pa*.*) alexandri*, *Lu*. *(Lu*.)almerioi*, *P*. *(La*.) ariasi*, *L*. *(Lu*.*) atunesi*, *P*. *(Ad*.) balcanicus*, *P*. *(Ad*.*) brevis*, *P*. *(Ad*.) chinensis*, *Lu*. *(Lu*.) cruzi*, *Lu*. *(Pf*.) evansi*, *Lu*. *(Lu*.*) forattenii*, *P*. *(Ad*.*) halepensis*, *P*. *(La*.) kandelakii*, *P*. *(Ad*.*) Kyreniae*, *P*. *(La*.) langeroni*, *P*.*(La*.*) longicuspis*, *P*. *(Ad*.) longiductus*, *Lu*. *(Lu*.) longipalpis*, *P*. *(La*.*) major s*.*l*.*, *Lu*. *(Lu*.*) migonei*, *Lu*. *(N*.*) olmeca olmeca*, *Lu*. *(V*.*) ovallesi*, *P*. *(La*.*) perfiliewi s*.*l*.*, *P*. *(La*.) perniciosus*, *Lu*. *(Lu*.*) pseudolongipalpis*, *Lu*. *(Lu*.*) sallesi*, *P*. *(Ad*.*) simici*, *P*. *(Ad*.) sichuanensis*, *P*. *(La*.) smirnovi*, *P*. *(La*.) tobbi*, *P*. *(Ad*.) turanicus*, *P*. *(La*.) wui*	North Africa, Mediterranean countries (Europe and North Africa), Southeast Europe, Middle East, Central Asia, North, Central and South America (Brazil, Venezuela, Bolivia, Mexico)	[157,180, 181,182]
		*L*. *major*	OW	CL	X	X			*P*. *(Syn*.*) ansarii*, *P*. *(P*.*) bergeroti*, *P*. *(Par*.) caucasicus*, *P*. *(P*.) duboscqi*, *P*. *(Par*.*) mongolensis*, *P*. *(P*.) papatasi*, *P*. *(P*.) salehi*	Central and North Africa, Middle East, Central Asia	[183,184, 218]
		*L*. *mexicana* (syn. of *L*. *pifanoi*)	NW	CL, DCL	X	X			*Lu*. *(D*.*) anthophora*, *Lu*. *(Hel*.) ayacuchenisis*, *Lu*. *(C*.*) christophei*, *Lu*. *(V*.*) columbiana*, *Lu*. *(Lu*.*) cruciata*, *Lu*. *(Lu*.*) diabolica*, *Lu*. *(N*.*) flaviscutellata*, *Lu*. *(Lu*.*) gomezi*, *Lu*. *(Lu*.*) longipalpis*, *Lu*. *(Lu*.*) migonei*, *Lu*. *(N*.) olmeca olmeca*, *Lu*. *(V*.) ovallesi*, *Lu*. *(Psy*.*) panamensis*, *Lu*. *(Ps*.*) shannoni*, *Lu*. *(N*.*) ylephiletor*	United States of America, Ecuador, Peru, Venezuela	[56,94, 185,186, 187,188,189]
		*L*. *tropica* (syn. of *L*. *killicki*)	OW	CL, VL	X	X			*P*. *(La*.*) aculeatus*, *P*. *(Ad*.) arabicus*, *P*. *(Par*.*) chabaudi*, *P*. *(La*.) guggisbergi*, *P*. *(Syn*.) rossi*, *P*. *(Pa*.) saevus*, *P*. *(Par*.) sergenti*	Central and North Africa, Middle East, Central Asia, India	[81,190,191]
		*L*. *turanica*	OW	_	X				*P*. *(P*.*) papatasi*	Central Asia, South Mongolia, Iran	[192,193]
		*L*.*venezuelensis*	NW	CL	X	X			*Lu*. *(Lu*.*) lichyi*, *Lu*. *(N*.*) olmeca bicolor*, *Lu*. *(Ps*.*) panamnsis*, *Lu*. *(V*.*) spinicrassa*	Northern South America, Venezuela	[19]
	***VIANNIA*** (growth in the hindgut of sandfly)	*L*. *braziliensis*	NW	CL, MCL	X	X			*Lu*. *(N*.*) anduzei*, *Lu*. *(Psy*.*) ayrozai*, *Lu*. *(Ps*.) carrerai*, *Lu*. *(V*.*)columbiana*, *Lu*. *(Ps*.) complexa*, *Lu*. *(Lu*.*)cruciata*, *Lu*. *(Lu*.*)edwardsi*, *Lu*. *(Pi*.) fischeri*, *Lu*. *(Lu*.) gomezi*, *Lu*. *(N*.*) intermedia*, *Lu*. *(Lu*.*) lichyi*, *Lu*. *(Ps*.) llanosmartinsi*, *Lu*. *(Lu*.*) longipalpis*, *Lu*. *(Lu*.) migonei*, *Lu*. *(N*.)neivai*, *Lu*. *(Lu*.)nuneztovari anglesi*, *Lu*. *(V*.) ovallesi*, *Lu*. *(Psy*.)panamensis*, *Lu*. *(Psy*.*)paraensis*, *Lu*. *(V*.*)pescei*, *Lu*. *(Lu*.*) pessoai*, *Lu*. *(V*.*)pia*, *Lu*. *(X*.) shawi*, *Lu*. *(V*.) spinicrassa*, *Lu*. *(Psy*.*) squamiventris*, *Lu*. *(Hel*.*) tejadai*, *Lu*. *(Lu*.*) townsendi*, *Lu*. *(Lu*.*) trinidadensis*, *Lu*. *(N*.*) trapidoi*, *Lu*. *(N*.*) umbralitis*, *Lu*. *(N*.) whitmani*, *Lu*. *(Ps*.) wellcomei*, *Lu*. *(N*.) ylephiletor*, *Lu*. *(Lu*.*) youngi*, *Lu*.*(Psy*.) yucumensis*	Western Amazon basin, South America, Brazil,Bolivia, Peru Guatemala, Venezuela	[19,64,172,174,194,195,196,197,198]
		*L*. *guyanensis*	NW	CL, MCL	X	X			*Lu*. *(N*.) anduzei*, *Lu*. *(Hel*.) ayacuchensis*, *Lu*. *(N*.*) flaviscutellata*, *Lu*. *(V*.*) longiflocosa*, *Lu*. *(Psy*.*) llanosmartinsi*, *Lu*. *(Lu*.*) migonei*, *Lu*. *(V*.*) ovallesi*, *Lu*. *(N*.) shawi*, *Lu*. *(N*.) umbratilis*, *Lu*. *(N*.) whitmani*	Northern South America, Bolivia, Brazil, French Guiana, Suriname	[38,172,174,199,200]
		*L*. *lainsoni*	NW	CL	X	X			*Lu*. *(V*.) nuneztovari anglesi*, *Lu*. *(N*.*) olmeca bicolor*, *Lu*. *(T*.) ubiquitalis*, *Lu*. *(N*.*) whitmani*	Brazil, Bolivia, Peru	[201]
		*L*. *lindenbergi*	NW	CL	X	X			*L*. *(Lu*.*) atunesi*	Brazil	[39]
		*L*. *naiffi*	NW	CL	X	X			*Lu*. *(Psy*.*) amazonensis*, *Lu*. *(Ps*.) ayrozai*, *Lu*. *(Lu*.*) gomezi*, *Lu*. *(Psy*.*) paraensis*, *Lu*. *(Ps*.) squamiventris*, *Lu*. *(N*.*) trapidoi*	Brazil, French Guyana	[172,199,202]
		*L*. *panamensis*	NW	CL, MCL	X	X			*Lu*. *(T*.*) cruciata*, *Lu*. *(N*.*) flaviscutellata*, *Lu*. *(Lu*.) gomezi*, *Lu*. *(Hel*.) hartmanni*, *Lu*. *(Mig*.*) migonei*, *Lu*. *(V*.*) ovallesi*, *Lu*. *(Psy*.) panamensis*, *Lu*. *(Hel*.*) sanguinaria*, *Lu*. *(V*.*) spinicrassa*, *Lu*. *(N*.) trapidoi*, *Lu*. *(N*.*) umbratilis*, *Lu*. *(N*.*) ylephiletor*, *Lu*. *(N*.) yuilli*	Central and South America, Brazil, Panama, Venezuela, Colombia	[19,174,203,204,205]
		*L*. *peruviana*	NW	CL, MCL	X	X			*Lu*. *(Hel*.) ayacuchensis*, *Lu*. *(Hel*.*) noguchii*, *Lu*. *(Hel*.) peruensis*, *Lu*. *(Hel*.*) tejadai*, *Lu*. *(V*.) verrucarum*	Peru, Bolivia	[19,172,174]
		*L*. *shawi*	NW	CL	X	X			*Lu*. *(N*.) whitmani*	Brazil	[201]
		*L*. *utingensis*	NW	Unknown				X	*Lu*. *(Vi*.*) tuberculata*	Brazil	[206]
	***SAUROLEISHMANIA*** (growth in the hindgut o*f* sandfly)	*L*. *adleri*	OW	_			X		*S*. *(Si*.*) clydei*, *S*. *(S*.*) dentata*	Iran, Kenya	[104,207,208, 217]
		*L*. *agamae*	OW	_			X		*P*. *(Pa*.*) caucasicus*, *P*. *(P*.*) papatasi*, *S*. *(S*.*) sintoni*	Eastern Mediterranean, Palestine, Lebanon, Israel, Turkmenistan	[104,209,210,211]
		*L*. *ceramodactyli*	OW	_			X		*P*. *(Pa*.*) caucasicus*, *P*. *(P*.*) papatasi*, *S*. *(S*.*) sintoni*	Eastern Mediterranean, Iraq, Sudan, Turkmenistan	[104,209]
		*L*. *chameleonis*	OW	_			X		Unknown	Egypt, Israel	[104,210]
		*L*. *davidi*	OW	_			X		Unknown	Central Africa	[104,210]
		*L*. *gulikae*	OW	_			X		Unknown	Turkmenistan	[213]
		*L*. *gymnodactyli*	OW	_			X		*P*. *(Pa*.*) caucasicus*, *S*. *(Si*.*) clydei*, *S*. *(S*.*) dentata*, *P*. *(P*.*) papatasi*, *S*. *(S*.*) sintoni*	Sudan, Iran, Turkmenistan	[102,150,209,212]
		*L*. *helioscopi*	OW	_			X		Unknown	Turkmenistan	[214]
		*L*. *hemidactyli*	OW	_			X		Unknown	India	[104]
		*L*. *henrici*	OW	_			X		Unknown	Martinique island (?)	[104]
		*L*. *hoogstraali*	OW	_			X		*S*. *(Si*.*) clydei*	Sudan, Senegal	[104,215,216]
		*L*. *nicollei*	OW	_			X		Unknown	Turkmenistan	[214]
		*L*. *phrynocephali*	OW	_			X		Unknown	Turkmenistan	[214]
		*L*. *platycephala*	OW	_			X		Unknown	Tanzania	[128]
		*L*. *senegalensis*	OW	_			X		*S*. *(S*.*) dubia*	Senegal	[104,213,215]
		*L*. *sofieffi*	OW	_			X		Unknown	Russia (Caspian Sea)	[102]
		*L*. *tarentolae*	OW	_			X		*S*. *(S*.*) antennata*, *S*. *(S*.*) minuta*, *P*. *(P*.*) papatasi*	North Africa, Malta, Sudan, Algeria, Italy, France, Malta	[24,104,175,211,219,220, 221]
		*L*. *zmeevi*	OW	_			X		*S*. *(S*.*) arpaklensis*, *P*. *(P*.*) papatasi*	Turkmeistan	[209,222]
		*L*. *zuckermani*	OW	_			X		Unknown	Sudan, South Africa	[223]
		*L*. *(S*.*) sp*. *I*	OW	_			X		Unknown	Pakistan	[210]
		*L*. *(S*.*) sp*. *II*	OW	_			X		Unknown	Pakistan	[210]
**UNCLEAR**	***L*. *enrietti* complex**	*L*. *enrietti*	NW	_	X				*Lu*. *(Lu*.*) gasparviannai*, *Lu*. *(Lu*.*) gomezi*, *Lu*. *(Pf*.*) monticola*	Brazil	[174,229]
		*L*. *martiniquensis*	NW, OW	CL, VL	X	X			Unknown	Martinique, Thailand	[230,231]
		“*L*. *siamensis*”	OW, NW	VL, CL	X	X			*S*. *(Ne*.*) gemmea*	Central Europe, Thailand, USA	[232,233,234]
		“*L*. *australiensis*”	Australia	_	X^M^				Midges	Australia	[228]
	***PARALEISHMANIA***	*L*. *colombiensis*	NW	CL, VL	X	X			*Lu*. *(Lu*.*) gomezi*, *Lu*. *(Hel*.) hartmanni*, *Lu*. *(Psy*.*) Panamensis*	Colombia	[101,224]
		*L*. *deanei*	NW	_	X^P^				*Lu*. *(Vi*.*) furcata*	South America, Brazil	[225]
		*L*. *equatorensis*	NW	_	X^S^				*Lu*. *(Hel*.*) hartmanni*	Ecuador	[226]
		*L*. *herreri*	NW	_	X^S^				*Lu*. *(Ps*.*) shannoni*, *Lu*. *(N*.*) trapidoi*, *Lu*. *(N*.*) ylephiletor*	Costa Rica	[149]
		*L*. *hertigi*	NW	_	X^P^				*Lu*. *(Psy*.*) chagasi*, *Lu*. *(Psy*.*) claustrei*, *Lu*. *(Psy*.*) davisi*, *Lu*. *(Psy*.*) squamiventris*	Panama, Costa Rica	[174,227]

*: Proven vector, Ad.: *Adlerius*, C.: *Coromyia*, CL: Cutaneous Leishmaniasis, DCL: Diffuse Cutaneous Leishmaniasis, Eu.: *Euphlebotomus*, Hel.: *Helcocyrtomyia*, L.: *Leishmania*, La.: *Larroussius*, Lu.: *Lutzomyia*, Mig.: *Migonei*, N.: *Nyssomyia*, Ne.: *Neophlebotomus*, P.: *Phlebotomus*, Pa.: *Paraphlebotomus*, Pf.: *Pifanomyia*, Pi: *Pintomyia*, Ps.: *Psathyromyia*, Psy.: *Psychodopygus*, S.: *Sergentomyia*, Si.: *Sintonius*, Sy.: *Synphlebotomus*, T.: *Tricholateralis*, V.: *Verrucarum*, Vi.: *Viannamyia*, VL: Visceral Leishmaniasis, X^M^: Mammal (Marsupials), X^P^: Mammal (Porcupines), X^R^: Mammal (Rodent), X^S^: Mammal (sloth)

First attempts at the classification of *Leishmania* were monothetic Linnean classifications that were proposed between 1916 and 1961, based on extrinsic characters only (Table 1). An early *Leishmania* classification was suggested by Nicolle in 1908, which separated *L*. *infantum*, the etiological agent of Mediterranean visceral leishmaniasis, from *L*. *donovani*, the causative agent of Indian kala azar. Then, Biagi proposed the separation of various New World *Leishmania* species [94] (see Table 1). In 1964 [95], Adler discussed the difficulties in accepting a clinically based taxonomy, as leishmaniasis may demonstrate the same clinical symptoms but by two different *Leishmania* species, e.g., visceral leishmaniasis with cutaneous symptoms. The most intensive and extensive investigations on these parasites were carried out in the Turkmenian USSR (reviewed by Belova, [96]). Other attempts to classify mammalian *Leishmania* in the traditional way (that is, by naming and defining species and subspecies) were presented by Lainson and Shaw [97,98] and Bray et al. [99]. In 1976 [100], Vickerman proposed the recognition of four species complexes within the genus: the *donovani* complex, the *tropica* complex, the *mexicana* complex and the *braziliensis* complex (adapted later partially by Lainson and Shaw). In 1979 [101], Lainson and colleagues described three sections of *Leishmania*, according to the intravectorial development of the parasite: Hypopylaria (saurian *Leishmania* developing in the hindgut), Peripylaria (developing in the hindgut and pylorus), and Suprapylaria (all development anterior to the pylorus). In 1982 [102], the Russian researcher Saf'janova proposed separation of *Leishmania* infecting lizards from other *Leishmania* species that infect mammals, and she proposed the name *Sauroleishmania* for these species [103]. The saurian *Leishmania* species were then assigned to a separate genus *Sauroleishmania* by Killick-Kendrick et al. [104]. A milestone for *Leishmani*a classification was the system presented by Lainson and Shaw in 1987, who divided the genus *Leishmania* into two subgenera, *L*. (*Leishmania*) for the section Suprapylaria and *L*. (*Viannia*) for the section Peripylaria. In the early 1970s, intrinsic characteristics (immunological, biochemical, and molecular) of *Leishmania* were identified and used to develop new classification systems. Isoenzyme electrophoresis, developed in the 1970s, has been widely used as a typing system and was accepted over decades as the gold standard for identification and is still a valuable tool as a reference technique for parasite characterization. Since the 1980s, Adansonian phenetic classification, based on the multiple similarity-weighted characters (absence of hierarchy) applied simultaneously (polythetic classification) without an a priori hypothesis, has been employed for *Leishmania* classification. Subsequently, phylogenetic analyses revealed a parental relationship between different species of *Leishmania*. The phenetic and, especially, the cladistic classification confirmed the majority of the taxonomic groups previously established through Linnean classifications, particularly that of Lainson and Shaw [19]. Pioneering phenetic classifications based on izoenzymes have been proposed by Moreno et al. [105], Thomas-Soccol et al. [106], and Cupolillo et al. [107] for the New World and by Lanotte et al. [108] and Le Blanq et al. [109] for the Old World. Rioux et al. [110] combined all New and Old World taxa in one classification system. Several of these authors also applied a phylogenetic concept of classification [111] that is based on the concepts of monophyletism, parsimony of changes, and nonconvergence of characters [106,112]. The concordance between these classifications mutually validated both the extrinsic (geographic distribution, associated clinical syndrome, and developmental features in the sandfly gut) and intrinsic (biochemical, immunological, and molecular markers) identification criteria applied. However, cladistic analyses allowed a more detailed analysis of some groups and led to the establishment of some new complexes of species (*L*. *infantum*, *L*. *turanica*, *L*. *guyanensis*). However, some of these complexes were later rejected by molecular data. In addition, these cladistic analyses led to the proposal to place previously separated species in the same complex (*L*. *guyanensis*, *L*. *panamensis*, *L*. *shawi*) [113].

Recently, a new classification for *Leishmania* has been proposed based on combined molecular data, which divides *Leishmania* species into two major phylogenetic lineages referred to as sections *Euleishmania* and *Paraleishmania* [114]. The section *Euleishmania* comprises four subgenera: *Leishmania* (type strain: *L*. *donovani*), *Viannia* (type strain: *L*. *braziliensis*), *Sauroleishmania* (type strain: *L*. *tarentolae*), and *L*. *enriettii* complex (type strain: *L*. *enriettii*). Section *Paraleishmania* includes *L*. *hertigi*, *L*. *deanei*, *L*. *herreri*, *L*. *equatorensis*, and *L*. *colombiensis* as well as the former *Endotrypanum* genus. Of this group, only *L*. *colombiensis* was found to be pathogenic to humans. The evolutionary history of the section *Paraleishmania* has not been yet resolved, and it is so far a polyphyletic clade within the genus *Leishmania*. Based on izoenzyme data, the genus *Leishmania* was shown to be monophyletic, but inference of its origin and evolution is complicated by its disjunct geographic distribution [106]. Especially with respect to the position of *Endotrypanum*, with its intraerythrocyte developmental stage as well as distinct morphology (epimastigote or trypomastigote form) within section *Paraleishmania*, as shown by molecular data, this remains questionable and has to be carefully reevaluated. The subgenus *Viannia* is restricted to the Neotropics, while the subgenus *Leishmania* occurs in both the New and Old World. Fifty-three named species (without synonyms, including all five subgenera and complexes: *Leishmania*, *Viannia*, *Sauroleishmania*, *L*. *enrittii* complex, and *Paraleishmania*) are recognized, 29 of which are present in the Old World, 20 in the New World, three species (“*L*. *siamensis*,” *L*. *martiniquensis*, and *L*. *infantum*) in both Old and New World, and one species in Australia (“*L*. *australiensis*”). Among these recognized species, 20 (without synonyms) are known to infect humans (updated information from Maroli et al. [39]).

Synonymy was shown for several species using molecular typing, e.g., *L*. *tropica* (syn. *L*. *killicki*) [117,118,119] and *L*. *donovani* (syn. *L*. *archibaldi*) [120,121,122]. Synonymy was also suggested for *L*. *mexicana* (syn. *L*. *pifanoi*) and *L*. *amazonensis* (syn. *L*. *garnhami*). However, in all published studies, only a few representatives for these synonyms have been included, and they should be studied using an adequate sampling strategy. It was also shown by multilocus microsatellite typing (MLMT) that one species (*L*. *infantum*/*L*. *chagasi*) was only recently (ca. 500 years ago) brought from the Old World (namely Portugal) to the New World and that it found a suitable vector there [123,124]. For a number of species, the phylogenetic status is not yet resolved (species or subspecies or even synonyms), mainly because of the limited number of included isolates, e.g., for *L*. *amazonensis*, *L*. *garnhami*, *L*. *pifanoi*, *L*. *venezuelensis*, *L*. *aristidesi*, *L*. *forattinii*, *L*. *arabica*, *L*. *utingensis* (represented by only a single sample), *L*. *lindenbergi*, *L*. *enrietti*, and those belonging to the *Paraleishmania* section. Moreover, molecular data based mainly on *hsp70* [125] proved the existence of only nine monophyletic groups. These groups might represent distinct species, and several other species should be treated as subspecies within these main groups, which was also confirmed by MLMT studies, e.g., for *L*. *braziliensis* and *L*. *peruviana* as subspecies, *L*. *donovani* and *L*. *infantum* as subspecies, *L*. *guyanensis*, *L*. *shawi*, and *L*. *panamensis* as subspecies, *L*. *mexicana* and *L*. *amazonensis* as subspecies, *L*. *tropica* and *L*. *aethiopica* as subspecies, etc. [126]. However, not all known species have been included in these studies, especially for the *L*. *mexicana* complex.

In conclusion, molecular data based on sequences of different targets and on MLMT do not support the concept of species complexes presented by Lainson and Shaw [19,127], and the classification should be revised, including both suppression of several species and also downgrading some species to the level of subspecies. Ongoing whole-genome sequencing and SNP analysis as well as further analysis by multilocus sequence typing (MLST) and MLMT and an adequate sampling and inclusion of representatives of all species (with sufficient numbers of isolates from different areas of distribution) will contribute to further improvement of the classification of the *Leishmania* genus.

*Sauroleishmania* was originally described by Ranque in 1973 [103] as a separate genus. It includes 19 named and two unnamed species (*L*. [*S*.] sp. I, *L*. [*S*.] sp. II; Telford [210]), according to Ovezmukhammedov and Saf'janova [213], Killick-Kendrick et al. [104], and Telford [128], without specifying their taxonomic positions. Among these, ten species were considered as valid by Ovezmukhammedov and Saf'janova (Fig 1) [213]. They [213] also reported one species as *L*. (*S*.) sp. without any additional information about its descriptor (author) and taxonomic position. During the 1980s, *Leishmania* that infect lizards were placed in a new genus, *Sauroleishmania*, which was also primarily based on the use of extrinsic characters [104]. In 1986 [129], Saf’janova proposed that *Leishmania* species diverged from *Leptomonas* and that such parasites were present in primitive sandflies during the Mesozoic period. This idea was supported later by molecular data [85,130]. The two subgenera that encompass *Leishmania* infecting mammals were regarded as having been separated by continental drift during the Mesozoic, and it was suggested that *Sauroleishmania* developed only in the Old World because the presence of the sandfly vectors for these parasites is strictly restricted to the Old World [129].

The *L*. *enriettii* complex and related parasites form a well-supported monophyletic group (*L*. *enriettii* complex) that most likely represents a new subgenus (Pothirat et al. [115]; Kwakye-Nuako et al. [116]). The only two formally described and named members of this group are *L*. *enriettii*, described in 1948 and repeatedly isolated from domestic guinea pigs, and *Leishmania martiniquensis*, described in 2014 as a causative agent of human diseases. Another three members that have been accommodated into the *L*. *enriettii* complex are: (i) never formally described "*L*. *siamensis*" from human patients; (ii) unnamed species sometimes called "*L*. *australiensis*" from Australia marsupials, most likely transmitted by midges; and (iii) very recently (2015) introduced unnamed *Leishmania* species from human cases in Ghana. At the moment, the names of “*L*. *siamensis*” and “*L*. *australiensis*” are not taxonomically valid names. For this, these names have been used in this paper with quotation marks.

The *Endotrypanum* genus belonging to the *Paraleishmania* group is known as a parasite of sloths that is transmitted by *Lutzomyia* species in Central and South America. These parasites are found within the erythrocytes of the *Choloepus* and *Bradypus* sloth genera. Only two species, *Endotrypanum schaudinni* and *E*. *monterogeii*, have been described in this genus [131]. The parasites that have been obtained through the in vitro culture of infected blood from sloths and from *Lutzomyia* sandfly guts are promastigotes that are indistinguishable from *Leishmania* promastigotes. Sloths also serve as a reservoir of *L*. *braziliensis*, *L*. *guyanensis*, *L*. *herreri*, *L*. *equatoriensis*, and *L*. *panamensis*, which are transmitted by sandfly vectors. They could be one of the first vertebrate hosts in which the dixenous life cycle of *Leishmania* could have emerged.

## *Leishmania* Fossil Evidence

*Leishmania* belongs to the phylum Kinetoplastida, which is likely related to the phylum of Euglenids [132]. Both of these groups belong to the eukaryotic supergroup Excavata, for which fossil evidence suggests emergence during the Ordovician [133]. *Leishmania* might have originated during the Mesozoic, prior to the separation of Gondwana [106]. The first *Leishmania* fossil record was *Paleoleishmania proterus*, a digenetic *Leishmania* species associated with a blood-filled female of the sandfly *P*. *burmitis* in Burmese fossil amber (Cretaceous, 100 MYA) (Table 3) [134]. Within the alimentary canal of this sandfly, amastigotes (*n* = 20), promastigotes (*n* = 393), and paramastigotes (*n* = 64) of digenetic leishmanial trypanosomatids were observed. The observation of these different parasitic stages in the alimentary tract of the insect suggests that their presence was likely the result of a blood meal and that they were multiplying within the midgut. The blood cells were later identified as being of reptilian origin. They also described the development of putative amastigotes within whitish, spherical-to-oval vacuoles associated with some blood cells. The second fossil of *Paleoleishmania* species described was *P*. *neotropicum*, which was found in Dominican fossil amber (20 MYA). A large number of promastigotes (*n* = 20) and amastigotes (*n* = 20) were found in the gut of *L*. *adiketis*. Additionally, four promastigotes, two paramastigotes, and several amastigotes of *P*. *neotropicum* were found in the proboscis of *L*. *adiketis*. The presence of amastigotes demonstrated the digenetic life cycle of *P*. *neotropicum*, as this parasitic life stage is considered to be present only in the vertebrate host, and no monogenetic flagellates are known to colonize sandflies.

The kingdom Animalia appeared 700 MYA, and the first *Leishmania* host ancestor likely also appeared at this time. In this period, the Earth was covered by water with a lower oxygen concentration [135]. The definitive hosts for primitive *Leishmania* may therefore have been reptiles or primitive mammals. It was initially suggested that the *Leishmania* genus originated in the Palaeocene, following the emergence of the first placental mammals. The ancestors of *Leishmania* emerged during the Ordovician [130,136], while winged insects appeared during the Carboniferous (300 MYA), and the first hematophagous winged insect appeared during the Cretaceous (140 MYA) [137]. The separation between primitive *Phlebotomus* and *Lutzomyia* arose approximately 200 MYA [138]. While trypanosomatids were present during the Palaeozoic, free-living forms were likely more diverse in the past than today. In this period, the *Leishmania* ancestor was separated into *Sauroleishmania* (reptile-infecting *Leishmania*) and the current *Leishmania* genus (mammal-infecting *Leishmania*) [139]. Subsequently, the division of *Leishmania* into *L*. (*Leishmania*) and *L*. (*Viannia*) occurred approximately between 54 to 25 MYA, after the separation of Africa from South America [140]. Geologically, the Earth experienced a cooling and drying period (1.5–2.5 MYA). The grassland biomes required for the development of the earliest murid rodents likely shifted towards the equator and the tropical forest biomes [141]. Along with their required biome, sigmodontine rodents (Rodentia: Muridae: Sigmodontinae) travelled across the Panamanian land bridge into South America.

The observation of sandfly larvae that develop in habitats containing trypanosomatid flagellates led to the hypothesis that sandflies host monoxenous trypanosomatids, and that these flagellates were carried through the pupal into the adult stage. This corresponds with the fact that *Leishmania* parasites evolved originally from *Leptomonas* monoxenous trypanosomatids [85], which are rarely transmitted to mammalian hosts, including humans [130]. The transmission of flagellates by an adult sandfly to a vertebrate host, establishing a continuing cycle between the vector and vertebrate species, likely occurred before the appearance of placental mammals during the Palaeocene. Thus, the appearance of placental mammals appears to have occurred after the appearance of the currently known *Leishmania* vectors, i.e., *Phlebotomus* and *Lutzomyia* species. Hence, the vector, mammalian host, and fossil record all suggest that leishmaniasis may have been established during the Palaeocene (65–31 MYA).

## Palaearctic Origin of *Leishmania*

**Fig 2:**
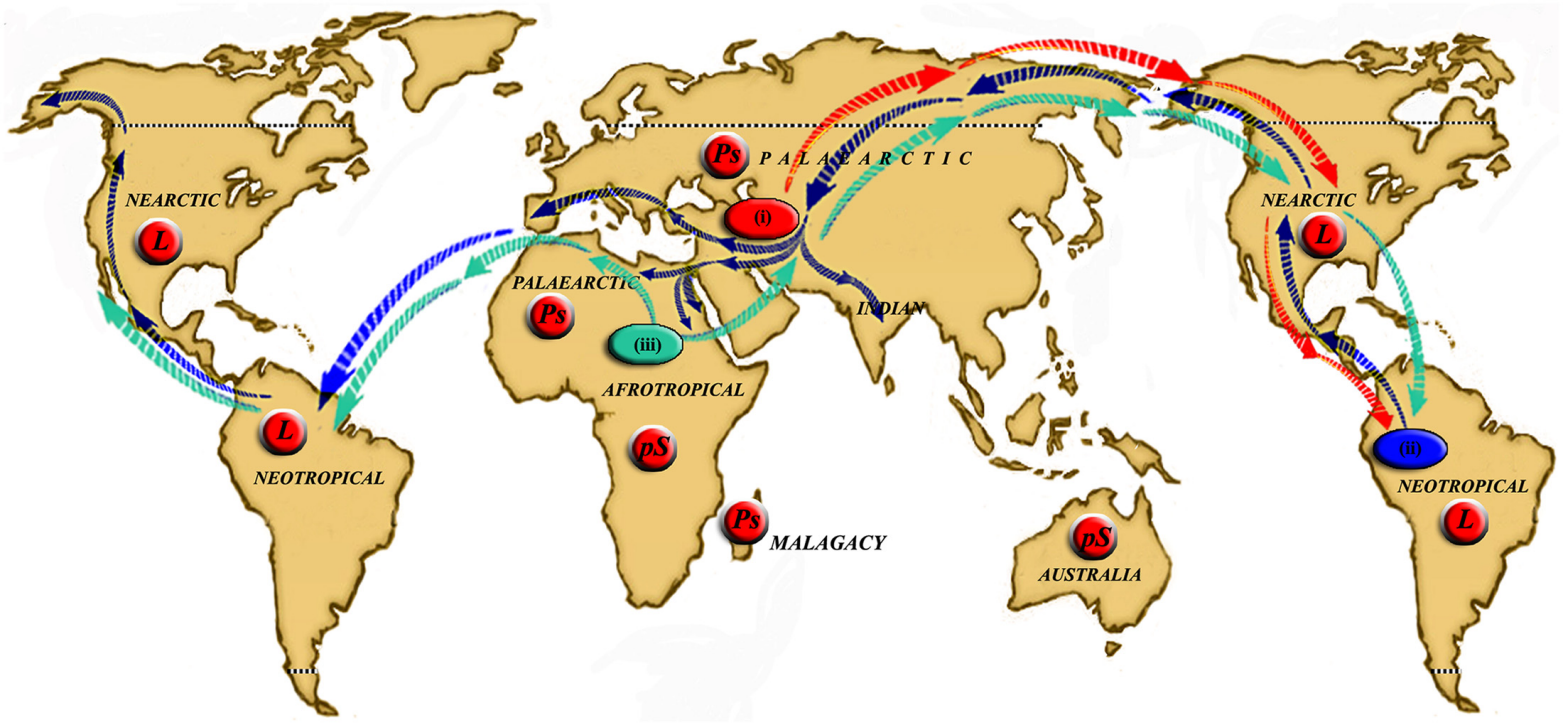
Possible routes of dissemination of *Leishmania*. (i). Red arrow: Palearctic origin of *Leishmania* (Lysenko [142], Kerr [136,144], Kerr et al. [145]). (ii) Blue arrow: Neotropical origin of *Leishmania* (Croan et al. [150], Noyes [149], Noyes et al. [83], Lukeš et al. [146]). (iii) Green arrow: Neotropical/African origin of *Leishmania* (Momen and Cupolillo [139]). Distribution of medically important sandflies is highlighted by red symbols. *L*: *Lutzomyia*, *P*: *Phlebotomus*, *S*: *Sergentomyia*, *PS*: Relative density and diversity of *Phlebotomus* as compared to *Sergentomyia*.

A Palaearctic origin of the genus *Leishmania* was proposed by Lysenko in 1971 [142]. Fossil evidence indicates that both phlebotomine sandflies and murid rodents originated in the Palaearctic [5,143], making it likely that *Leishmania*, along with its vectors and reservoirs, could have evolved in the Palaearctic during the Cenozoic period and dispersed to the Nearctic during the Oligocene (Eocene), when the Bering land bridge was intact. These species then dispersed into the Neotropics across the Panamanian land bridge during the Pliocene, when the climate was sufficiently warm to permit further dispersal of *Leishmania* (Fig 2) [82,142,144,145].

Molecular analyses of *Leishmania* strains coming from various Old World endemic areas suggest that *L*. *donovani* and *L*. *infantum*, which are responsible for VL, likely diverged approximately 1 MYA. *Leishmania donovani* subsequently invaded India and Africa [146], and 500 years ago, *Leishmania infantum* was transported to South America and was named *L*. *chagasi*, which is now considered to be synonymous with *L*. *infantum* [146–148].

*P*. *proterus* found in sandflies fed with reptile blood in the Palaearctic during the Cretaceous period led to the hypothesis that reptiles were likely the original hosts of *Leishmania*. *Sauroleishmania* may have then diverged from *L*. *(Leishmania)* in the Old World as a consequence of its adaption to reptiles. *Sauroleishmania* could have originated in Cretaceous reptiles residing in the Palaearctic region and subsequently declined during the Cenozoic period because of cooling of the Earth, as mammals radiated. Thus, the successful establishment of *Leishmania* appears to have been assisted by first infecting reptiles. This evolutionary scenario is supported by some molecular data and the numerous reptilian trypanosomes that are transmitted by today’s sandflies. The infections then shifted to the murid rodents, which are now the most significant reservoirs of *Leishmania* strains causing CL. Murid rodents likely appeared in the Palaearctic during the Oligocene era and then dispersed across the Bering land bridge to Nearctic regions during the Eocene era. Mice and rats from the New World evolved in the Nearctic ecozone before crossing the Panamanian land bridge to the Neotropics during the Pliocene, after which they underwent a rapid radiation, leading to the introduction of parasites to caviomorth rodents, sloths, armadillos, and anteaters [136,141,144]. All of these species act as reservoirs and play an important role in the persistence and dispersal of the parasites because of their relatively long lifespan compared with sandflies [136,141]. The origin and dispersion of murid rodents has been taken as essential evidence that *Leishmania* originated in the Palaearctic region. Around this time, phlebotomine species ancestral to both *Phlebotomus* and *Lutzomyia* adapted to feed on rodents instead of reptiles, likely because their burrows offer humidity and shelter from cold for both rodents and sandflies. The fossil record indicates that the phlebotomine sandfly ancestor evolved in the Palaearctic (Cretaceous, 120 MYA) and that *Phlebotomus* also evolved in the Palaearctic (Eocene, Baltic amber), and *Lutzomyia* diverged from *Phlebotomus* (Oligocene, Mexican amber) after the breaking of the Bering land bridge [136,141,145].

## Neotropical Origin of *Leishmania*

In 1998 [149], Noyes suggested a Neotropical origin of *Leishmania* during the Palaeocene or Eocene period (36–46 MYA). Subsequently, the parasites invaded the Nearctic ecozone via the Panamanian land bridge and the Palaearctic via the Bering land bridge during the Miocene. The greater diversity observed among New World *Leishmania* species compared with those from the Old World provides some circumstantial evidence arguing for a Neotropical origin of *Leishmania* [19,150]. Nevertheless, if this hypothesis is true, then *Sauroleishmania* might have evolved later during the Miocene, either in the Nearctic or the Palaearctic area, as a result of adaptation to reptiles [149]. Sloths (Xenarthra) might have served as the first vertebrate reservoirs of *Leishmania* in the Neotropics. Also, it has been suggested that a number of monogenetic and digenetic trypanosomatids can grow in the rectal glands of marsupials. After adaptation to rodents during the Eocene, infected porcupines would have carried the parasites across the Panamanian land bridge to the Nearctics and across the Bering land bridge to the Palaearctic during the Miocene in an unspecified mammalian reservoir (Fig 2) [83,149,150].

Climate change, in combination with the topographic diversity found in the Central and South America, has certainly played a role in the vicariance of the sigmodontine rodents and their accelerated speciation. The cricetids (sigmodontines) encompass approximately 40 genera and more than 200 species that evolved within approximately 2.5 MYA [141]. A similarly rapid rate of evolution is observed in New World *Leishmania* [141,151].

## Neotropical/African Origin of *Leishmania*

According to this theory, the genus *Leishmania* is divided into two sections: *Euleishmania* (*Leishmania* and *Viannia* subgenera and *Sauroleishmania*) and *Paraleishmania* (*L*. *hertigi*, *L*. *deanei*, *L*. *colombiensis*, *L*. *equatorensis*, and *L*. *herreri*) [114,139]. It is also speculated that the separation of Gondwana in the Mesozoic resulted in the evolution of the *Leishmania* genus into *Leishmania* and *Sauroleishmania* in Africa, and *Viannia* and *Paraleishmania* in South America [139]. The origin and the evolution of *Leishmania* would have been related to the origin of humans in eastern Africa, with *Leishmania* following the dynamics of the human population in the Palaearctic (Asia, Africa, and Europe) ecozone. An African origin of *Leishmania* was emphasized by Momen and Cupolillo [139], based on the importance of the origins of its vectors and reservoirs as evidence for this hypothesis and citing the restricted habitat of *Arvicanthis* rodents and *Phlebotomus* sandflies in Africa. According to this hypothesis, the Old World *Leishmania* species (e.g., *L*. *donovani*/*L*. *infantum*, *L*. *tropica*, *L*. *major*, and *L*. *aethiopica*) exhibit an African origin. *L*. *aethiopica* is present only in the Ethiopian and Kenyan highlands. Because of its restricted geographical distribution, it is reasonable to assume an African origin for this species as well as for the other *L*. *(Leishmania)*–hyrax systems that occur in Africa [128]. The origin of humans from eastern Africa suggests that *Leishmania* species with anthroponotic transmission, i.e., *L*. *tropica* and *L*. *donovani*, may also have originated in eastern Africa (Fig 2) [152].

## Relationship between Sandflies and *Leishmania*

The term “coevolution” was first used to demonstrate a particular type of relationship between *Leishmania* and sandfly species in the Old World [147]. *Leishmania* and sandflies have survived over many millions of years under selective pressure, depending on natural ecological changes. A close relationship has been demonstrated between some sandfly and *Leishmania* species, such as *L*. *major* and *P*. *papatasi*. This longstanding evolutionary history of *Leishmania* and sandflies has resulted in a similar distribution. However, there is not always a clear distinction between coevolution and certain other concepts, such as coassociation (meaning that the transmission cycle exhibits a distinctive landscape epidemiology), interaction (the molecular and immunological relationship between the sandfly midgut and the parasite’s external surface), or vector–parasite cospeciation or co-cladogenesis [37]. Most *Leishmania* parasites are more restricted regarding the range of sandfly vectors that can transmit them than in the range of mammalian hosts/reservoirs they are able to infect, suggesting a much closer coevolutionary relationship with sandflies than with their vertebrate hosts, although it is sometimes difficult to interpret this coevolutionary relationship [153]. For example, there is a specific relationship between *P*. *papatasi* and *L*. *major* because of the presence of specific midgut receptors [154], and these two species show strong distribution sympatry. Nevertheless, such high specificity of *Leishmania* for its sandfly vector appears to be restricted to *P*. *papatasi* or *P*. *duboscqi* and *P*. *sergenti*. However, the appearance of *Leishmania* interspecies hybrids might have consequences in terms of specificity and transmission efficiency [155,156].

The incrimination of sandflies as proven or potential vectors of *Leishmania* is a controversial and debated matter. Five criteria stated by Killick-Kendrick [104] are required to incriminate a particular sandfly species as a vector, which include the observation of corresponding epidemiological data, feeding behaviour of the sandflies on the animal intermediate host, the isolation of promastigote parasites from the sandflies, the occurrence of the complete life cycle of the parasite in its putative vector, and experimental transmission of the parasite through the bite of the infected species. Since the 1990s, with PCR invention and advances in molecular parasitology, molecular evidence was added to the mentioned criteria, and reports regarding the presence of *Leishmania* DNA in various sandfly species have dramatically increased. Nevertheless, according to the above-mentioned criteria, the presence of *Leishmania* DNA within sandflies should certainly not be considered to be a sufficient criterion to incriminate a sandfly species as a proven vector. Further evidence highlighting the presence of metacyclic promastigotes within the insect’s gut as well as demonstration of the insect’s capacity to retransmit *Leishmania* are essential criteria that need to be investigated to indicate the vectorial competence of sandflies. Approximately 166 species have been reported to be proven or potential vectors of different *Leishmania* species in the Old and New World (Table 4). Among these species, 78 are reported as the proven vectors of *Leishmania*. In the Old World, *Leishmania* are transmitted by sandflies belonging to the *Phlebotomus* genus (49 species, 31 are reported as proven), while *Sauroleishmania* are transmitted by sandflies of the *Sergentomyia* genus. In the New World, *Leishmania*, *Viannia*, and *Endotrypanum* species are transmitted by sandflies belonging to the *Lutzomyia* genus (118 species, 47 are reported as proven). Among the above-mentioned sandfly vectors, seven are involved in the transmission of *L*. *major*, seven in the transmission of *L*. *tropica*, 31 in the transmission of *L*. *infantum*, and nine in the transmission of *L*. *donovani*. New World sandflies (genus *Lutzomyia*) are involved in the transmission of different species (see Table 4, updated information from various publications). The stronger restriction of vectors to cutaneous *Leishmania* species than to vectors of either the visceralizing *donovani*/*infantum* group [147] or *L*. *(Viannia)* [19] provides support for the hypothesis that cutaneous species evolved first.

**Fig 3:**
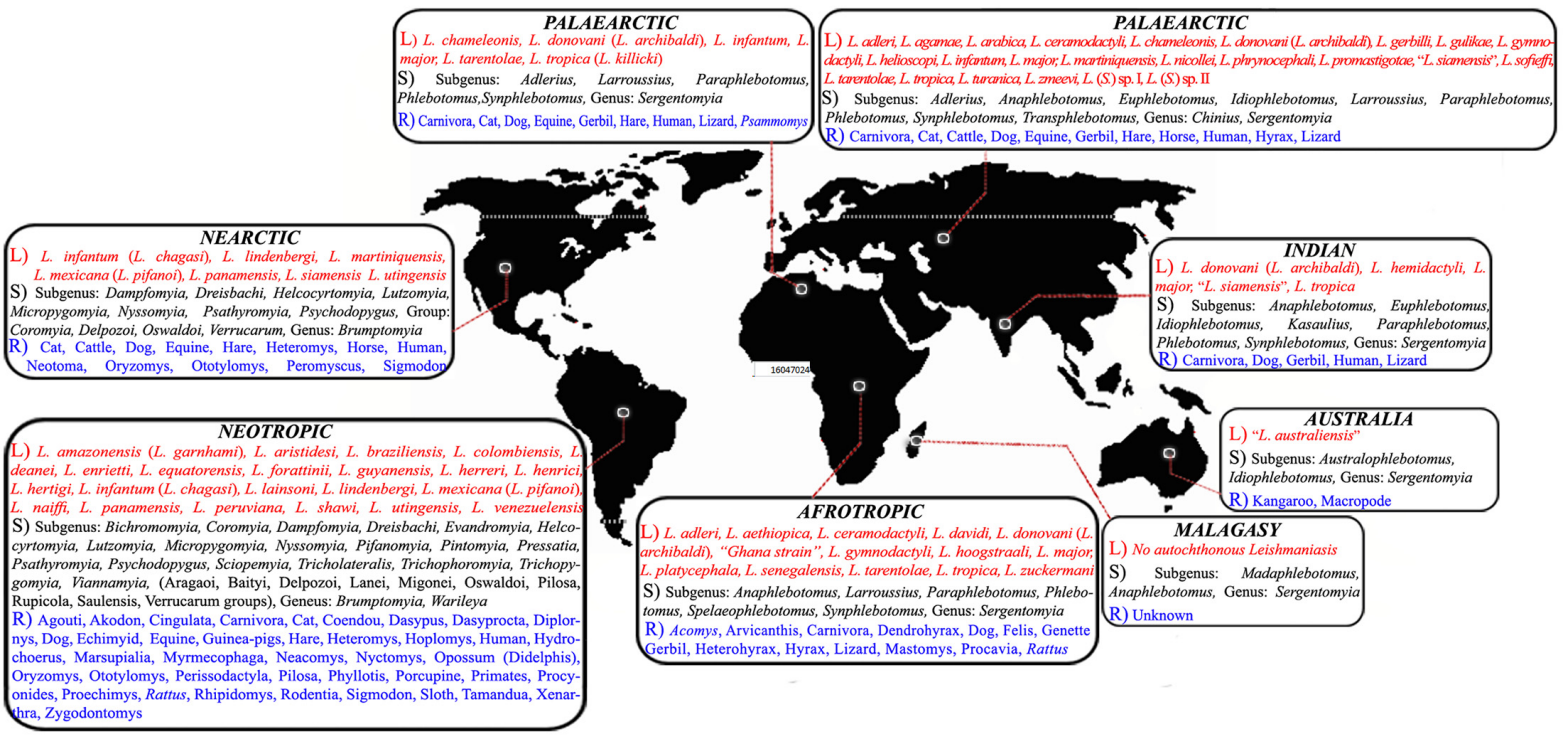
Geographical distributions of various *Leishmania* spp.; sandflies and animal reservoirs in the Old and New World. L: *Leishmania* (species), S: Sandfly (genus or subgenus), R: Reservoir (genus or family).

Cutaneous leishmaniasis (CL) is a vector-borne zoonotic disease, involving various wild rodents and humans as vertebrate hosts and different sandfly species as vectors playing a role in *Leishmania* transmission. In the Old World, a large majority of CL cases are geographically restricted to the arid and semiarid areas of the North, Central sub-Saharan, and East African regions; the Near East and Middle East; and Central Asia and India. New World CL occurs in tropical and subtropical areas of Mexico and Central and South America. The *Leishmania* species responsible for CL differ between the Old and New World. In the Old World, the etiological agents of CL include *L*. *tropica*, *L*. *major*, and *L*. *aethiopica*, whereas New World CL is caused by parasites of the *L*. *mexicana* complex (*L*. *mexicana*, *L*. *amazonensis*, *L*. *pifanoi*, *L*. *garnhami*, and *L*. *venezuelensis*) or the subgenus *Viannia* (*L*. *braziliensis*, *L*. *guyanensis*, *L*. *panamensis*, *L*. *naiffi*, *L*. *shawi*, *L*. *lainsoni*, and *L*. *peruviana*). In the Old World, the proven vectors of CL are mainly classified in the subgenera *Phlebotomus* and *Paraphlebotomus*, even though some species of the *Adlerius* and *Larroussius* subgenera are thought to be vectors of parasites causing Old World CL [81,157]. In the New World, the main vectors of CL belong to the subgenera *Nyssomyia*, *Psychodopygus*, *Lutzomyia* s.str., and *Verrucarum* (Fig 3) (Table 4).

Diffuse cutaneous leishmaniasis (DCL) was first reported in Kenya in 1969. This disease is an anergic variant of localized CL, in which lesions are disseminated. The causative agent is *L*. *aethiopica*, which is transmitted by *P*. *pedifer* and *P*. *longipes*. Nevertheless, DCL caused by *L*. *amazonensis*, transmitted by *Lutzomyia*-group *Olmeca* in the New World, has also been reported.

Mucocutaneous leishmaniasis (MCL), or espundia, occurs exclusively in South America, showing a greater incidence in Peru, Bolivia, Paraguay, Ecuador, Colombia, and Venezuela. *L*. *braziliensis* (*Viannia* subgenus) is the main causative agent, and to a lesser extent, *L*. *guyanensis*, *L*. *panamensis*, and *L*. *amazonensis* are also known to be responsible for MCL in this region. The vectors of this disease mainly belong to the subgenus *Psychodopygus* (e.g., *L*. (*Ps*.) *wellcomei*) [158].

Visceral leishmaniasis (VL) is usually a systemic disease that affects internal organs, particularly the spleen, liver, and bone marrow. *L*. *donovani* and *L*. *infantum* are the agents responsible for Old World VL, whereas *L*. *chagasi* (synonym with *L*. *infantum*) is responsible for New World VL. Some VL cases caused by *L*. *tropica* or *L*. *amazonensis* have also been reported [159]. The main VL vectors belong to the *Euphlebotomus*, *Larroussius*, and *Synphlebotomus* subgenera [160], but some species of the *Adlerius* and *Paraphlebotomus* subgenera have also been reported as vectors of *L*. *infantum* and *L*. *donovani*. The vectors involved in the transmission of New World VL belong to the *Lutzomyia* sensu stricto, *Migonemyia*, *Nyssomyia*, *Pifanomyia*, *Psychodopygus*, and *Verrucarum* subgenera (Fig 3) [161].

## Discussion and Conclusion

Phlebotomine sandfly systematics, particularly at the supraspecific level, have always been controversial [34,53]. Originally, this family was composed of a single genus: *Phlebotomus* Rondani. In 1948, Theodor proposed subdivision of the sandfly family into four genera: *Phlebotomus* and *Sergentomyia* in the Old World and *Lutzomyia* and *Brumptomyia* in the New World. A "stable" classification of the phlebotomine sandflies was proposed in 1977 by Lewis and colleagues [14], who retained the well-known family, subfamily, and genus names. It was also proposed that the subgenera and species groups be used as a model to put forward a new proposal. A “flexible” classification was proposed by Ready and colleagues in 1980 [162]. These researchers challenged the “stable” classification through a comparative analysis of characters that were described as “exclusive” characters for their proposed genera, e.g., *Phlebotomus*, *Sergentomyia*, *Brumptomyia*, *Warileya*, and *Psychodopygus*, but no such characters were found for *Lutzomyia*. The absence of unique characters for the genus *Lutzomyia* is certainly the weakest point in their comparative character analysis. New discoveries in later years led to the erection of new subgenera or genera. One of the difficulties in sandfly classification concerns the position of sandfly species at the genus or subgenus level. There is no general agreement regarding the definition of some groups at the genus or subgenus level. *Idiophlebotomus* in *Phlebotomus*, as well as *Parrotomyia*, *Rondanomyia*, and *Grassomyia* in *Sergentomyia* were classified by Quate and Fairchild [163] at the subgenus level, whereas Abonnenc [164] considered *Idiophlebotomus* to be genus and *Sergentomyia* to be a subgenus. Abonnenc and Minter [165] did not include *Parvidens* as a subgenus of *Sergentomyia*, whereas Abonnenc [164] considered *Parvidens* to be a subgenus of the *Phlebotomus* genus. Lewis [5] declined to recognize generic status for *Spelaeophlebotomus* and *Idiophlebotomus*, whereas Artemiev and Neronov [166] considered them at the genus level. Similarly, for New World sandfly species, Young and Duncan [8] classified *Bichromomyia*, *Dampfomyia*, *Deanemyia*, *Evandromyia*, *Expapillata*, *Martinsmyia*, *Micropigomyia*, *Migonemyia*, *Nyssomyia*, *Pintomyia*, *Psathyomyia*, *Psychodopigus*, *Trichophoroymyia*, *Trichopigomyia*, and *Viannamyia* to be subgenera of the *Lutzomyia* genus, whereas Galati et al. [66] elevated these groups to the genus level. These conflicts in classification are mainly due to (i) differences or variations in the criteria and the methods used for classification, such as criteria that are now considered to be outdated or scarce, e.g., the presence of erected or recumbent abdominal setae; (ii) morphological similarities between species and some uncertainty in species identification, such as the existence of cryptic or sibling species and the similarity of morphological characters among females that makes species identification dependent on male characters (e.g., *Adlerius*); (iii) the inadequacy of the reported species descriptions; and (iv) the massive increase in the number of sandfly species described. The construction of a well-supported phylogeny of the generic and subgeneric groups in the Phlebotominae subfamily will likely require a supermatrix analysis. This matrix must include molecular information on several nuclear genes combined with mitochondrial genes—as well as other criteria related to biology—and ecology, which has been successfully applied for the classification of the *Drosophilidae* family [167]. This type of analysis would provide a firmer basis for the classification of Phlebotominae sandflies, in addition to resolving the problem of the proposal of classifications suggested for the Old World and New World sandflies. Therefore, a more extensive molecular phylogenetic analysis, e.g., focussing on gene flow and the phenotypes of specimens, awaits the development of an accurate and valid protocol for sandfly classification.

A reliable taxonomy of *Leishmania* species will represent a keystone for biological and epidemiological research programs. There is still no universal agreement regarding the classification of *Leishmania*, especially concerning the criteria defined for species definition, or the method used to address phylogenetic classification. The greatest inconsistency concerns the assignment of *Leishmania* at the specific or subspecific level. Although the clustering of *Leishmania* at the subgeneric level and the definition of “complexes” in *Leishmania* classification have gained rather wide acceptance since being reported by Lainson and Shaw [98], there are still serious challenges in terms of the genus composition. Various molecular methods have been introduced to elucidate the taxonomy of *Leishmania*, though defining a *Leishmania* species or accepting all of the described species is still not straightforward. The currently accepted classification of *Leishmania* proposes the division of this genus into three subgenera: *Leishmania*, *Viannia*, and *Sauroleishmania*. Under this proposal, species that cannot be classified into any of these subgenera are included in the *Paraleishmania* section, such as yet-unclear-status *Leishmania* parasites. A question that remains open to debate is the position and classification of *Sauroleishmania*. Because this group is of low medical importance, there is little information about the reliability of its classification at present. Its placement in the *Leishmania* phylogeny therefore remains highly debated. Contradictorily, Kerr [144] proposed that the mammalian *Leishmania* evolved from lizard *Sauroleishmania* in the Palaearctic, whereas Noyes [149] controversially suggested that lizard *Sauroleishmania* evolved from mammalian parasites. This group has been placed both at the crown of the phylogeny [83,139,150] and at its root [136,144,145]. It appears more likely that the position of *Sauroleishmania* external to all *L*. (*Leishmania*) is a consequence of a faster rate of evolution in this subgenus, as suggested by a molecular phylogenetic analysis performed on the RNA and DNA polymerase genes [150]. Therefore, the systematic position of many *Leishmania* infecting reptiles remains unresolved. This difficulty in assigning a phylogenetic position is likely due to (i) the paucity of information about the life cycle of *Sauroleishmania*; (ii) the fact that all of the flagellates found in reptiles have been studied mainly at the light-optical level (except some submitted sequences in Genbank), without additional study methods being applied (serological, biochemical, and others), whereas some flagellates from reptiles belong to *Trypanosoma* and are also transmitted by sand flies; and (iii) the existence of a priori notions that every flagellate detected in a reptile’s body should be attributed to *Leishmania* promastigotes without further study of their true identity. Therefore, to avoid any doubt in the classification of *Leishmania* as well as *Sauroleishmania*, emphasis on the exploration of new isolates via molecular biology and phylogenetic (DNA analysis) methods is suggested. Finally, to clarify the position of *Leishmania* species in this classification, it is proposed that assignment to major groups across the entire genus *Leishmania* should be based on gene sequences, which are remarkably congruent and uncontroversial. For classification within the major groups, more highly discriminatory markers, such as MLST markers, microsatellites, or genome-wide single nucleotide polymorphisms, are considered to be better suited.

Knowledge about the origin and dispersal of *Leishmania* will help us to more precisely understand the factors that have and will continue to influence the circulation of leishmaniasis, in relation to its etiological parasitic agents, the vectors that transmit them, and their reservoirs. The dissemination of *Leishmania* has followed the migration of its vectors and hosts together [168]. Concerning the origin of *Leishmania* species, several hypotheses have been proposed, which were described above. These hypotheses profit from significant fossil, molecular, ecological, and biochemical data supporting them. Nevertheless, the debate is still open. To gather more information to support hypotheses of the origin and evolution of *Leishmania*, more evidence must be considered. Such evidence will include the following:

- Molecular phylogenies: based on several independent genes that display different evolutionary constraints, e.g., the elongation factor (EF-1α), heat shock protein gene (*hsp70*), and glyceraldehyde dehydrogenase (GAPDH), SSU (small subunit of ribosomal DNAs), DNA Polymerase α (POLA), cytochrome b (cytb), cysteine proteases, RNA polymerase II large subunit, gp63, mini-exon, and internal transcribed spacer of rDNA (ITS) (at lower taxonomical level) and spliced leader (SL) genes. Some of these genes are single-copy, protein-coding genes and are therefore suitable candidates for studying the molecular systematics and phylogeny of *Leishmania* [169].
- Biogeographical and ecological evidence: geographical, ecological, and climatic aspects as well as geological periods of the Earth and the presence of natural environmental pressures or geographical barriers must be investigated to obtain insight into the origin, evolution, and dispersion of *Leishmania*. It is worth considering that the absence or emergence of geographical barriers, such as mountains, in the past few million years (or even today), has resulted in a wider or restricted distribution of *Leishmania* parasites and their sandfly vectors and animal hosts at a worldwide scale.
- Entomological evidence: considering that leishmaniasis is a vector-borne disease, it is of course essential to more precisely understand the origin and the evolution of sandfly vectors along with *Leishmania* development, considering their coevolution and sympatry in different periods of time.
- Mammalogical evidence: considering that leishmaniasis is a zoonotic disease, the origin, conservation, and dispersion of *Leishmania* is highly dependent on animal reservoirs.

Three hypotheses have been proposed concerning the origin of *Leishmania* (Fig 2). Kerr [144] proposed a Palaearctic origin of *Leishmania*, based on a study carried out by Lysenko in 1971 [142]. He used fossil evidence of mammalian taxa and sandflies previously reported by Nowak [143] and Lewis [5], respectively, to support his hypothesis. Nevertheless, this hypothesis has been proposed based on a biogeographical study, which must be tested against other independent datasets. In 2000 [144], based on biogeographical evidence, fossil records of mammals and sandflies, and ecological data, Kerr also proposed a revision of the *Leishmania*/*Sauroleishmania* clade, but the lack of an independent phylogenetic analysis undermined the reliability of this hypothesis. Several factors argue against a Neotropical origin of *Leishmania*. Based on this theory, (i) porcupines did not move from the Neotropic to the Nearctic, whereas the fossil record demonstrates that such migration occurred after the formation of the Panamanian land bridge during the Pliocene [143]; (ii) porcupines did not travel across the Bering land bridge; (iii) the use of nonmolecular evidence, such as data based on biogeography, epidemiology, ecology, and historical events, is controversial; and (iv) there is an inconsistency between the current classifications of phlebotomine sandflies and the proposed Neotropical origin of *Leishmania* as well as a discrepancy between a Palaearctic origin of the murid rodents and a Neotropical origin of the parasite [7,139,144]. The third hypothesis considers *Leishmania* to exhibit a Neotropical/African origin. Despite reported evidence, this theory does not consider human dispersion into the Neotropics [139]. Finally, based on this hypothesis, a serious question remains regarding the *Sauroleishmania* phylogeny at the crown of the phylogenic tree and the dispersal of *Leishmania* from Africa to the Neotropics before the separation of Pangaea when considering the lack of evidence concerning the presence of *Sauroleishmania* in the Neotropics.

The question about *Leishmania* evolution has classically been centred on two opposing theories related to the original host for *Leishmania* as a digenetic parasite; i.e., was the first host a vertebrate or an invertebrate? Such information will certainly help us to better understand the origin and factors that play an important role in *Leishmania* dispersion and therefore in the epidemiology of leishmaniasis. The Phlebotominae ancestor emerged in the Triassic period, before the appearance of *Leishmania* (Jurassic) and placental mammals (Palaeocene). This hypothesis is further supported by an SSU rRNA data analysis indicating that *Leishmania* diverged from a trypanosomatid line of monogenetic insect parasites [140]. The oldest fossil ancestors of the modern sandflies date from the Cretaceous period (120 MYA, Lebanon), followed by Burmese fossil amber (Cretaceous, 100 MYA). A gap of approximately 80 MYA is present from this Burmese fossil amber specimen until the next fossil found in Baltic amber (20 MYA), meaning that there is a serious gap in knowledge. According to the Burmese fossil amber specimen, ingested and free-living flagellates of *P*. *proterus* were found in habitats containing *P*. *burmitis* sandflies. In the Jurassic period, the reptiles were the predominant vertebrate fauna for many years. Despite their presence, there is no strong evidence, such as fossils, linking the sandfly lineage with ancient cold-blooded vertebrates. This absence or rarity of *Leishmania* in older reptiles suggests that sandflies with haematophagous habits were likely to be the first host of *Leishmania*. In addition, the greater range restriction of the sandfly vectors than the animal hosts of *Leishmania* parasites supports the much closer coevolutionary relationship of *Leishmania* and sandflies. Considering the above observations, it appears that monogenetic parasites of sandflies adapted to mammals some 90 MYA, giving rise to *Leishmania*. This adaptation likely took place during a period when mammals were diversifying into different orders during the separation of Africa and South America. Kerr [144] proposed a Palaearctic origin of *Leishmania*, suggesting that reptiles were the first vertebrate hosts of *Leishmania*, whereas Noyes [149] considered rodents to be the first vertebrate host. With the exception of the *Sauroleishmania* group, no human pathogenic *Leishmania* have been reported from reptiles. One the other hand, regarding some characteristics of sandflies, such as their restricted flight distance, short life cycle, slow larval development, and greater blood feeding preference for warm-blooded animals compared with cold-blooded species, it is assumed that these insects were the first host of *Leishmania*, but they have not played a major role in the *Leishmania* dispersion, particularly in regions that are unsuitable for sandfly survival. Hence, it is assumed that *Leishmania* were transferred by infected sandflies to local vertebrates, in which the parasite can survive for long period, after which the vertebrates, particularly the murid rodents, were the responsible for disease dispersion in the Old and New World. Muroids are a large superfamily of rodents. They have diversified into a large superfamily comprising over 1,500 species, including hamsters, gerbils, true mice, and rats as well as many other relatives. They now make up nearly one-third of all mammalian species, and they occupy a vast variety of habitats on every continent except for Antarctica. Comparison of the origin and distribution pattern of rodents proposed by Schenk et al. [170] (Steppan [171]) with the hypotheses of *Leishmania* appearance and dispersion suggests a close similarity in the distribution patterns of these groups, supporting the theory that they might be responsible for *Leishmania* dispersion in both the Old and New World.

## Concluding Remarks

The evolutionary relationship between sandflies and *Leishmania* has implications for leishmaniasis interventions and control. It is therefore necessary to obtain information on the origin of *Leishmania* and the Phlebotominae sandflies and their chronological history of coevolution. Understanding these evolutionary relationships between different *Leishmania* and sandfly species is of epidemiological importance for the future prediction of *Leishmania* transmission patterns.

**Key Learning Points**

- Understanding the current hypotheses of the origin and dispersion of *Leishmania* and sandflies, based on the available fossil evidence and molecular studies and the factors that play important role in these dispersions
- To have a knowledge about three-century history of sandflies and *Leishmania* classification as well as a complete description of *Leishmania* and sandfly fossils, with biological emergence date of each *Leishmania* and sandfly groups during different geographical periods from 550 million years ago until now
- An update of information on the current distribution and dispersion of different species of *Leishmania* (53 species), sandfly vectors (More than 800 species), and animal reservoirs in each geographical regions of Palearctic, Nearctic, Neotropic, Afrotropical, Oriental, Madagascar, and Australia
- A critical discussion on the different approaches that were used for *Leishmana* and sandfly classification, their advantages and disadvantages, their synonymy, and proposal of an updated classification for each species of *Leishmania* and sandfly
- Suggesting a complete list of the potential and proven sandfly vectors for each *Leishmania* species in the Old and New World

**Top Five Papers**

1. Lainson, R., Shaw, J. J. (1987) Evolution, classification and geographical distribution. In: W Peters, R Killick-Kendrick, editors. The Leishmaniases in Biology and Medicine, Academic Press, London, p. 1–120.
2. Cupolillo, E., Medina-Acosta, E., Noyes, H., Momen, H., Grimaldi, G. Jr. (2000) A revised classification for *Leishmania* and *Endotrypanum*. Parasitology Today, 16, 142–144.
3. Kerr, S. F. (2000) Palaearctic Origin of *Leishmania*. Memorias de Instituto Oswaldo Cruz, 95, 75–80.
4. Poinar, Jr. G. O. (2004) Palaeomyia burmitis (Diptera: Phlebotomidae), a new genus and species of Cretaceous sandflies with evidence of blood-sucking habits. Proceedings of the Entomological Society of Washington, 106, 598–605.
5. Lewis, D. J. (1982) A taxonomic review of the genus *Phlebotomus* (Diptera: Psychodidae). Bulletin of the British Museum (Natural History), 45, 121–209. http://www.sandflycatalog.org/pdfs/116803.pdf
