# Correction: A Historical Overview of the Classification, Evolution, and Dispersion of *Leishmania* Parasites and Sandflies

**DOI:** 10.1371/journal.pntd.0004770

**Published:** 2016-06-02

**Authors:** The PLOS Neglected Tropical Diseases Staff

Some information from the caption of Fig 1 is missing. Please see the complete, correct Fig 1 caption here.

**Fig 1:**
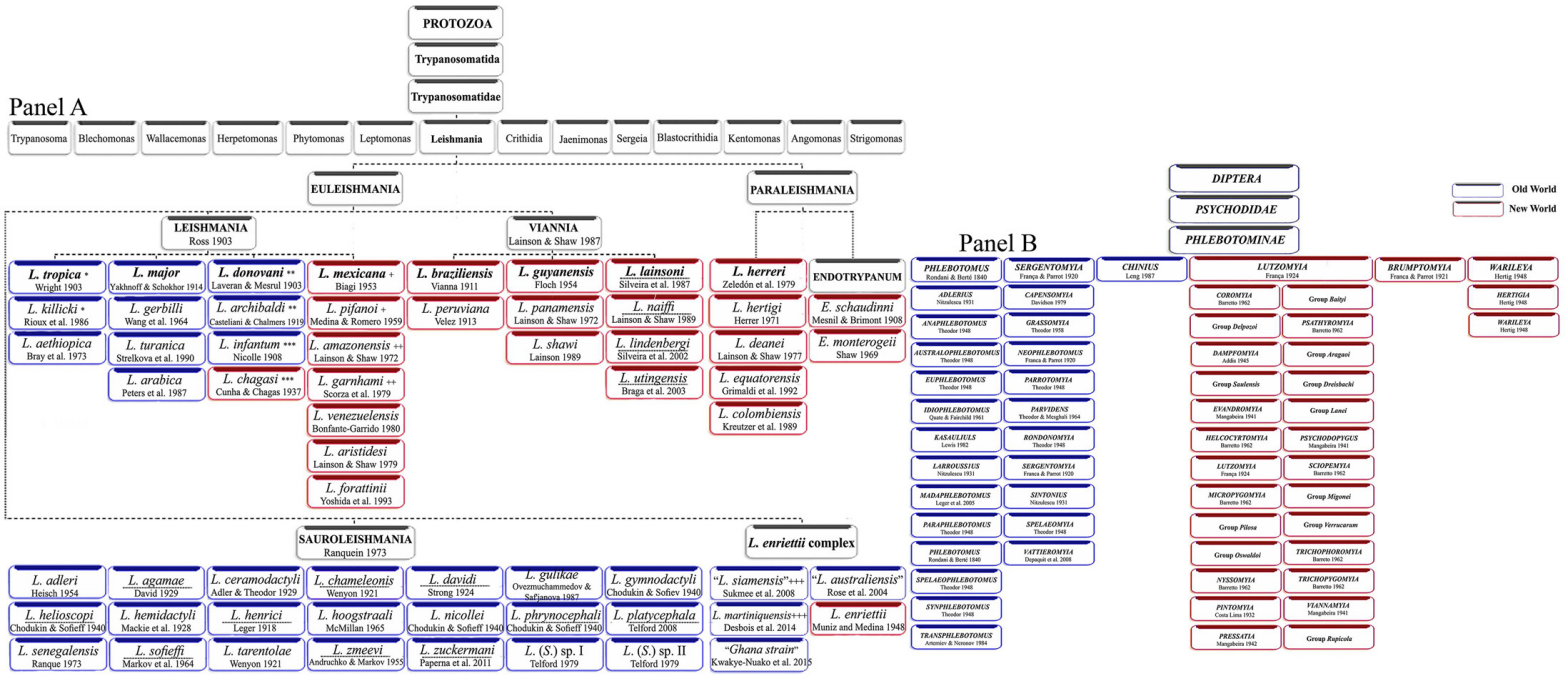
Updated classification of Leishmania and sandfly. Panel A. Classification of Leishmania species. *, +: Synonym; different numbers of star (*) and plus (+) signs mean which species name is the synonym for which original species, Underlined: No final classification. ″L. siamensis″ and L. martiniquensishave been found also in the New World. Leishmania names in quotationmark are unofficial names without formal descriptions. Panel B. Phlebotominae sandfly classification,according to Theodor [6,13], Quate and Fairchild [163], Theodor and Mesghali[22], Lewis [5], Leng [15], and Young and Duncan [8].

## References

[1] Akhoundi M, Kuhls K, Cannet A, Votýpka J, Marty P, Delaunay P, et al (2016) A Historical Overview of the Classification, Evolution, and Dispersion of *Leishmania* Parasites and Sandflies. PLoS Negl Trop Dis 10(3): e0004349 doi: 10.1371/journal.pntd.0004349 26937644 10.1371/journal.pntd.0004349 PMC4777430

